# Neutral lipid fatty acid composition as trait and constraint in Collembola evolution

**DOI:** 10.1002/ece3.3472

**Published:** 2017-10-16

**Authors:** Ting‐Wen Chen, Philipp Sandmann, Ina Schaefer, Stefan Scheu

**Affiliations:** ^1^ J. F. Blumenbach Institute of Zoology and Anthropology University of Göttingen Göttingen Germany; ^2^ Centre of Biodiversity and Sustainable Land Use University of Göttingen Göttingen Germany

**Keywords:** community phylogenetics, comparative method, functional traits, phylogenetic signal, springtails, trophic niche

## Abstract

Functional traits determine the occurrence of species along environmental gradients and their coexistence with other species. Understanding how traits evolved among coexisting species helps to infer community assembly processes. We propose fatty acid composition in consumer tissue as a functional trait related to both food resources and physiological functions of species. We measured phylogenetic signal in fatty acid profiles of 13 field‐sampled Collembola (springtail) species and then combined the data with published fatty acid profiles of another 24 species. Collembola fatty acid profiles generally showed phylogenetic signal, with related species resembling each other. Long‐chain polyunsaturated fatty acids, related to physiological functions, demonstrated phylogenetic signal. In contrast, most food resource biomarker fatty acids and the ratios between bacterial, fungal, and plant biomarker fatty acids exhibited no phylogenetic signal. Presumably, fatty acids related to physiological functions have been constrained during Collembola evolutionary history: Species with close phylogenetic affinity experienced similar environments during divergence, while niche partitioning in food resources among closely related species favored species coexistence. Measuring phylogenetic signal in ecologically relevant traits of coexisting species provides an evolutionary perspective to contemporary assembly processes of ecological communities. Integrating phylogenetic comparative methods with community phylogenetic and trait‐based approaches may compensate for the limitations of each method when used alone and improve understanding of processes driving and maintaining assembly patterns.

## 1 INTRODUCTION

Functional traits are measurable properties of species which influence their performance and fitness (Violle et al., 2007; Pey et al., 2014). They in part regulate the occurrence of species along environmental gradients and coexistence with other species in local communities (McGill, Enquist, Weiher, & Westoby, 2006; Ackerly & Cornwell, 2007; Adler, Fajardo, Kleinhesselink, & Kraft, 2013), where coexisting species may possess similar or different traits. Ecological traits have been assigned to two categories, α and β niche traits. While β niche traits determine species’ environmental tolerance, α niche traits relate to resource exploitation (Ackerly & Cornwell, 2007). Similar β niche traits but different α niche traits thus allow species to live under similar environmental conditions but utilize different resources (Silvertown et al., 2006).

Understanding evolution of traits in coexisting species helps to infer community assembly processes (Webb, Ackerly, McPeek, & Donoghue, 2002; Silvertown et al., 2006; Best & Stachowicz, 2013). Species’ traits may exhibit phylogenetic signal; that is, phylogenetically related species share similar traits derived from a common ancestor (Harvey & Pagel, 1991). In contrast, traits may evolve convergently, resulting in closely related species with dissimilar traits or distantly related species with similar traits (Cavender‐Bares, Ackerly, Baum, & Bazzaz, 2004). However, species’ traits may also be labile, that is, varying among species irrespective of phylogenetic relationships. Further, α and β niche traits may evolve in different ways and thus exhibit different phylogenetic signal: β niche traits are usually phylogenetically conserved, while α niche traits tend to be evolutionarily labile (Silvertown et al., 2006; Ackerly, Schwilk, & Webb, 2006; Best & Stachowicz, 2013). In this study, we measured phylogenetic signal in a ubiquitous trait of terrestrial microarthropods, that is, fatty acid composition.

Fatty acids (FAs) are major components of lipids, serving as a source of energy (i.e., neutral lipids) and structural components of cell membranes (i.e., phospholipids; Ruess & Chamberlain, 2010). Neutral lipid fatty acids (NLFAs) in animal fat deposits carry the signal of the diet. Some NLFAs are incorporated directly and unmodified from food resources and are useful as biomarkers to distinguish between major food resources in animals living in soil (Ruess & Chamberlain, 2010; Buse, Ruess, & Filser, 2013; Ferlian, Klarner, Langeneckert, & Scheu, 2015). These biomarker FAs include absolute bacterial biomarkers which are only synthesized by prokaryotes, such as a15:0, i15:0, 16:1ω5, 16:1ω7, i16:0, i17:0, cy17:0, 18:1ω7, and cy19:0, as well as relative biomarkers, such as plant biomarker 18:1ω9 and fungal biomarker 18:2ω6,9, which are found in high proportions when the consumer mainly feeds on plant or fungi, respectively. Thus, proportions of biomarker FAs imply α niche traits related to food resources. Other NLFAs, such as C20 polyunsaturated FAs 20:4ω6 and 20:5ω3, can be synthesized or modified from precursors by consumers (Chamberlain & Black, 2005; Ruess & Chamberlain, 2010). These FAs are essential for biosynthesis of other compounds such as prostaglandins and eicosanoids, which are associated with reproduction, immune response, and temperature regulation (Chamberlain, Bull, Black, Ineson, & Evershed, 2004; Chamberlain & Black, 2005; Haubert, Häggblom, Scheu, & Ruess, 2008). They thus represent β niche traits reflecting species environmental requirements.

Springtails (Hexapoda: Collembola) are among the most abundant soil invertebrates. They occur in virtually every terrestrial habitat reaching particularly high densities in soil and contribute to decomposition processes and nutrient cycling in terrestrial ecosystems (Rusek, 1998). They are ideal for exploring phylogenetic signal of FAs as they consume a wide range of food resources including detritus, roots and root exudates, bacteria, fungi, and algae (Hopkin, 1997). Fatty acid profiles have been used to identify food resources of Collembola and their association with different decomposition channels based on bacteria, fungi, or root exudates as basal resources (Ruess et al., 2005; Pollierer, Dyckmans, Scheu, & Haubert, 2012; Ferlian et al., 2015). Distinct FA profiles of different Collembola species suggest trophic niche differentiation among co‐occurring species (Chamberlain & Black, 2005; Ruess et al., 2007; Ferlian et al., 2015). This may be attributed to (1) taxonomic or evolutionary relationships between different phylogenetic groups (Chamberlain & Black, 2005), reflecting fixation of the physiology of species and their way of feeding over evolutionary time, and/or (2) ecological characteristics, such as life‐forms (eu‐, hemi‐, and epedaphic) or availability of food resources in a habitat (Ruess et al., 2007). Further, species assigned to different soil strata may have similar FA profiles, indicating the use of similar resources (Ferlian et al., 2015). Overall, FA composition of Collembola may be similar in closely related species (phylogenetic signal present) and/or determined by available resources and thus not related to phylogenetic affinity (phylogenetic signal absent).

In this study, we consider FA composition as a functional trait and analyze its phylogenetic signal using a comparative method (Harvey & Pagel, 1991; Freckleton, Harvey, & Pagel, 2002). Based on the α and β niche trait concept, we tested the following hypotheses: (1) C20 polyunsaturated FAs exhibit phylogenetic signal in Collembola, suggesting that closely related species have similar physiological attributes. (2) Food resource FA biomarkers in Collembola are phylogenetically independent as different species utilize different resources. We used two FA datasets: FA profiles measured in this study from 13 field‐sampled Collembola species and our data combined with published FA profiles of another 24 species (Table 1). We constructed a phylogenetic tree for all 37 Collembola species and measured phylogenetic signal in both FA datasets using two common comparative phylogenetic metrics, Blomberg's K (Blomberg, Garland, & Ives, 2003) and Pagel's lambda (Pagel, 1999; Freckleton et al., 2002).

**Table 1:** Taxonomy and collection habitat of the Collembola species used in this study

Phylogenetic group	Family	Species^a^	Habitat^b^	Reference
**Symphypleona**	**Sminthuridae**	***Allacma fusca***	**Arable field (1)**	**This study**
			**Forest (3)**	**This study**
			Forest	Chamberlain & Black (2005)
		***Sminthurus viridis***	**Arable field (1)**	**This study**
			**Grassland (5)**	**This study**
	**Bouletiellidae**	***Deuterosminthurus sulphureus***	**Arable field (1)**	**This study**
			**Grassland (2)**	**This study**
	Dicyrtomidae	*Dicyrtomina ornata*	Forest	Chamberlain and Black (2005)
			Forest	Ruess et al. (2005)
			Forest	Ruess et al. (2007)
		*Dicyrtomina sp. (D. saundersi)*	Forest	Ruess et al. (2005)
**Poduromorpha**	**Hypogastruridae**	***Ceratophysella denticulata***	**Forest (3)**	**This study**
			Forest	Ruess et al. (2005)
			Forest	Ruess et al. (2007)
			Forest	Ferlian et al. (2015)
		*Ceratophysella succinea* c	Grassland	Sechi et al. (2014)
		*Willemia anophthalma*	Arable field	Ngosong et al. (2009)
			Arable field	Ngosong et al. (2011)
	Brachystomellidae	*Brachystomella parvula* c	Grassland	Sechi et al. (2014)
	Neanuridae	*Neanura muscorum*	Forest	Ruess et al. (2005)
			Forest	Ruess et al. (2007)
		*Polyacanthella (Friesea claviseta)*	Arable field	Ngosong et al. (2009)
	Onychiuridae	*Onychiurus spp. (O. ambulans)*	Forest	Ruess et al. (2005)
		*Protaphorura armata*	Forest	Ferlian et al. (2015)
		*Protaphorura fimata (P. sp1)*	Arable field	Haubert et al. (2009)
		*Protaphorura spp. (P. sp2)*	Forest	Ruess et al. (2007)
**Tomoceridae**	**Tomoceridae**	***Pogonognathellus flavescens***	**Grassland (1)**	**This study**
			**Forest (6)**	**This study**
		*Pogonognathellus longicornis*	Forest	Chamberlain and Black (2005)
			Forest	Ruess et al. (2005)
			Forest	Ruess et al. (2007)
		***Tomocerus vulgaris***	**Forest (4)**	**This study**
		*Tomocerus baudoti*	Forest	Pollierer et al. (2012)
		*Tomocerus minor*	Forest	Chamberlain and Black (2005)
**Isotomidae**	**Isotomidae**	***Isotoma viridis***	**Arable field (4)**	**This study**
			**Grassland (4)**	**This study**
			Arable field	Ngosong et al. (2009)
			Arable field	Ngosong et al. (2011)
			Forest	Chamberlain and Black (2005)
		*Isotoma viridis* d	Grassland	Sechi et al. (2014)
		*Isotoma anglicana* d	Grassland	Sechi et al. (2014)
		*Desoria violacea*	Forest	Ruess et al. (2005)
			Forest	Ruess et al. (2007)
		*Folsomia quadrioculata*	Forest	Ruess et al. (2005)
			Forest	Ruess et al. (2007)
			Forest	Ferlian et al. (2015)
		*Isotomiella minor*	Forest	Ferlian et al. (2015)
		*Isotomurus palustris (I. fucicolus)*	Forest	Chamberlain and Black (2005)
		*Parisotoma notabilis*	Forest	Ruess et al. (2005)
			Forest	Ferlian et al. (2015)
**Entomobryoidea**	**Entomobryidae**	***Entomobrya muscorum***	**Grassland (2)**	**This study**
			**Forest (5)**	**This study**
			Forest	Ruess et al. (2005)
		***Entomobrya nicoleti***	**Grassland (2)**	**This study**
		*Entomobrya nivalis*	Forest	Ruess et al. (2005)
	**Lepidocyrtidae**	***Pseudosinella immaculata***	**Grassland (1)**	**This study**
		***Lepidocyrtus cyaneus***	**Arable field (4)**	**This study**
			**Grassland (5)**	**This study**
			Grassland	Sechi et al. (2014)
		***Lepidocyrtus lanuginosus***	**Arable field (2)**	**This study**
			**Grassland (4)**	**This study**
			**Forest (1)**	**This study**
			Forest	Pollierer et al. (2012)
			Forest	Ferlian et al. (2015)
		*Lepidocyrtus lignorum*	Forest	Ruess et al. (2005)
			Forest	Ruess et al. (2007)
		*Lepidocyrtus curvicollis (L. sp)*	Forest	Chamberlain and Black (2005)
	**Orchesellidae**	***Orchesella villosa***	**Arable field (8)**	**This study**
			**Forest (1)**	**This study**
			Forest	Chamberlain and Black (2005)
			Arable field	Haubert et al. (2009)
		*Orchesella flavescens*	Forest	Ruess et al. (2005)
			Forest	Ruess et al. (2007)

a The 13 species collected for this study are marked in bold. Name in parenthesis indicates the congeneric species used in the phylogeny constructed by sequences listed in Table S1.

b Number in parenthesis indicates replicate number in fatty acid measurements of field derived Collembola in this study (pooled for sites).

c Fatty acid data compiled using Poduromorpha in Sechi et al. (2014).

d *Fatty* acid data compiled using *Isotoma* spp. in Sechi et al. (2014).

## 2 METHODS

### 2.1 Sampling

Collembola were sampled from two sites near Göttingen, Germany, Deppoldshausen (51.575°N, 9.973°E) and Ossenfeld (51.548°N, 9.798°E). Each sampling site was composed of three adjacent habitats: arable field, pasture, and forest. In each habitat, five samples (1 m^2^, at least 5 m apart) were taken in June and July 2015. Collembola in arable fields and pastures were sampled using an aspirator, then immediately brought to the laboratory at the University of Göttingen, and sorted. Collembola in forests were extracted from leaf litter by heat (Kempson, Lloyd, & Ghelardi, 1963) at constant 35°C for 1 week. Collembola were sampled alive daily and immediately stored at −80°C until identification and lipid extraction. Species were identified according to Hopkin (2007). In total, sufficient biomass for FA extraction was obtained for 13 species.

### 2.2 Fatty acid analysis

Soil and organic matter was removed from the surface of each Collembola using a brush prior to FA extraction. Depending on body size of individuals and species, three to 36 individuals of the same species, and sample were pooled for one FA extraction. In total, 70 FA measurements were obtained, ranging from one to four replicates for each species per habitat and site.

NLFAs were extracted as described in Haubert, Häggblom, Scheu, and Ruess (2004). Neutral lipid fractions were dried at 50°C using a rotation vacuum concentrator (RVC 2‐25, Chris, Osterode am Harz, Germany). The lipid fractions were then saponified, methylated, and washed. The obtained FA methyl esters were transferred into vials, capped, and stored at −21°C until gas chromatography (GC) analysis. The gas chromatograph (Clarus 500, Perkin Elmer, Waltham, USA) was equipped with a flame ionization detector (PE‐5 capillary column, 30 m × 0.32 mm i.d., 0.25 mm film thickness, Perkin Elmer, Waltham, USA) and helium as carrier gas. The analysis program followed Ferlian and Scheu (2014). FA methyl esters were identified by comparing retention times of samples and standard mixtures comprising unbranched and branched FA methyl esters.

### 2.3 Collembola phylogeny

In addition to the above 13 Collembola species, published NLFA data were available for 24 additional species (Table 1). A phylogeny of all 37 Collembola species, spanning 12 families, was inferred based on a supermatrix (3,053 bp) composed of 18S and 28S rRNA, cytochrome oxidase subunit I (COI) and Histone H3 genes using MrBayes 3.2.4 (Ronquist et al., 2012). *Callibaetis* (Insecta: Ephemeroptera), *Machilis* (Insecta: Archaeognatha) and Zygentoma (Insecta) were used as outgroups. The resulting tree was transformed to an ultrametric tree by assuming a strict clock model using the function *chronos* implemented in the R package “ape” (Paradis, Claude, & Strimmer, 2004). This tree was then used in the phylogenetic signal measurement. For more details of phylogenetic inference, see Appendix S1.

### 2.4 Statistical analysis

For our field data, rare FAs present in only single measurement and FAs contributing less than 1% of total FAs were eliminated from the analyses. The remaining FAs were summed to 100%, and the proportions of single FAs were logit‐transformed using the function *logit* in the R package “car” (Fox & Weisberg, 2011). To test for differences in FA compositions between Collembola species and habitats, multivariate analysis of variance (MANOVA) and discriminant function analysis (DFA, function *lda* implemented in the R package “MASS”; Venables & Ripley, 2002) were used, with sites and habitats set as error terms in the model, followed by ANOVA with Holm's adjusted *p*‐values (Holm, 1979). For the FAs showing significant differences between Collembola species, Tukey's honestly significant difference (HSD) test was conducted. Fatty acid profiles of species were also explored using eigen decomposition principle components analysis (PCA). Species mean logit‐transformed FA proportions were calculated and then multiplied by the eigenvectors based on a covariance matrix using the species mean. Individual observational logit‐transformed FA proportions were multiplied by the same eigenvectors to examine intraspecific variation. Principle components (PCs) were selected if the variance explained by each axis was more than predicted by a broken stick model. Pearson's correlation coefficients of FAs and PCs were calculated using function *cor.test* in R with Holm's *p*‐value adjustment.

Three types of FA data were used to measure phylogenetic signal: (1) species mean scores on the PC axes, irrespective of site and habitat; (2) species mean proportion of individual FAs; (3) species mean values of FA indices, including sums of bacterial FAs, plant‐to‐fungal FA marker ratios (P:F ratio), bacterial‐to‐fungal FA marker ratio (B:F ratio), bacterial‐to‐plant FA marker ratio (B:P ratio), Unsaturation Index (UI; Haubert et al., 2004), sums of saturated FAs (SFAs), monounsaturated FAs (MUFAs), polyunsaturated FAs (PUFAs) and C20 PUFAs, and ratio of unsaturated‐to‐saturated FAs (U:S ratio). Phylogenetic signal was detected and quantified using both Blomberg's K (Blomberg et al., 2003) and Pagel's lambda (Pagel, 1999; Freckleton et al., 2002). These two metrics assume a Brownian motion model of trait evolution, that is, variance in trait values is directly proportional to branch length of a given phylogeny (Pagel, 1999; Blomberg et al., 2003). Both methods were used because they have different sensitivities in detecting phylogenetic signal for traits evolved with various strengths of Brownian motion and for trees with different size (Münkemüller et al., 2012). Phylogenetic signal analyses were conducted using the function *phylosig* implemented in the R package “phytools” (Revell, 2012). Standard errors of FA measurements were considered in Blomberg's K statistics (Ives, Midford, & Garland, 2007). Significance tests were carried out by randomizing species on the phylogeny 10,000 times, to test whether trait values show phylogenetic signal or not (i.e., *H*_0_ = 0). In case of significant *K*‐values of traits, the observed *K*‐value was further compared with 5,000 simulated *K*‐values to test whether phylogenetic signal was significantly different from the level expected under Brownian motion evolution model (i.e., *H*_0_ = 1; Revell, Johnson, Schulte, Kolbe, & Losos, 2007). Simulations of trait values were conducted using the function *fastBM* in the R package “phytools” (Revell, 2012). Lower and higher phylogenetic signal than predicted by a Brownian motion model was defined as a *K*‐value in the 0.025 and 0.975 quantiles of the log‐transformed simulated *K*‐values, respectively. All *p*‐values in phylogenetic signal measurement were adjusted using Benjamini & Hochberg's method (Benjamini & Hochberg, 1995). Phylogenetic signal of FAs was accepted only when both Blomberg's K and Pagel's lambda were significant.

As a small phylogenetic tree (13 species in our field‐sampled dataset) may lack power to detect phylogenetic signal (Freckleton et al., 2002; Blomberg et al., 2003; Münkemüller et al., 2012), FA phylogenetic signal was also measured using a combined dataset comprising data of our field‐sampled Collembola and published FA data (Table 1). Mean FA proportions were calculated for each species at each site and habitat for our FA data. Data from the literature were compiled at species level for each treatment or site by extracting the published mean values or recalculating original data provided by the authors. Due to inconsistency of FAs measured in different studies, only biomarker FAs, C20 unsaturated FAs, and saturated FAs 16:0 and 18:0 were included. Unavailable values of these FAs in literature data were replaced by zero assuming that they were not reported due to being present in trace amounts only. Fatty acids contributing less than 1% of total FAs and those occurring in only one sample were eliminated. The remaining FAs were summed to 100% and logit‐transformed, resulting in a final dataset of 37 species and 149 data points for phylogenetic signal measurements. Principle components and phylogenetic signal in species mean scores on PCA axes, mean proportion of individual FAs, and FA indices were analyzed as above.

## 3 RESULTS

### 3.1 Fatty acid composition of Collembola

**Figure 1:**
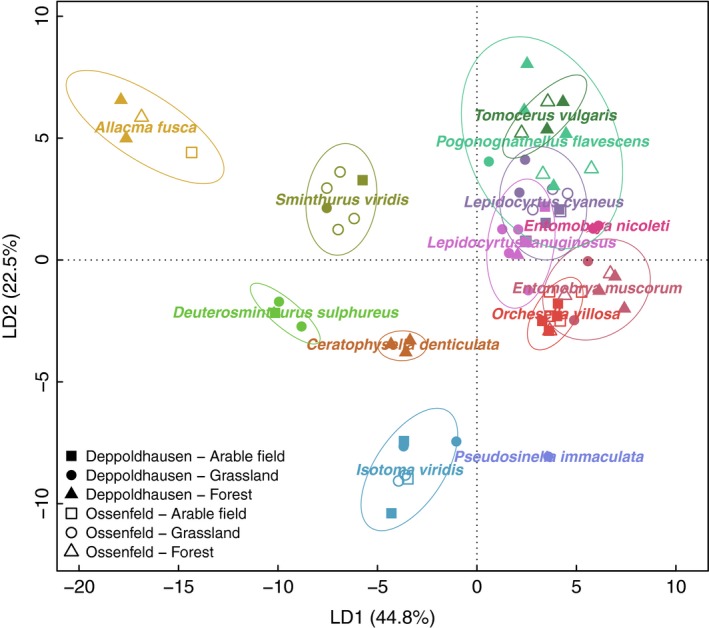
Discriminant function analysis of fatty acid profiles of 13 field‐sampled Collembola species. Ellipses represent confidence ranges at *p *=* *.05

Thirty‐two FAs were identified from the 13 field‐sampled Collembola species (Table S2). Frequent FAs (occurring in >30 of the 70 measurements) were 18:1ω9, 18:2ω6,9, 16:0, 18:0, 20:5ω3, 20:4ω6, 16:1ω7, 14:0, and 18:1ω7. Overall, the lipid composition of Collembola predominantly differed between species, whereas the effect of habitat was not significant (MANOVA, *F*_384,288_ = 1.65, *p *<* *.001 for species and *F*_256,160_ = 1.26, *p *=* *.058 for habitat). The DFA plot clearly separated the FA profiles between different species (Figure 1). *Allacma fusca*,* Deuterosminthurus sulphureus*,* Sminthurus viridis*,* Ceratophysella denticulata,* and *Isotoma viridis* were separated from the remaining species along the first two axes. The proportions of individual FAs differed among species (Tables S2, S3). Fatty acid 18:1ω9, a predominant FA in Collembola, was lower in *I. viridis* (12.4%), while it contributed 26.5%–42.2% to total FAs in all other species. Another major FA, 18:2ω6,9, was highest in the three Symphypleona species, *S. viridis* (37.6%), *A. fusca* (34.9%), and *D. sulphureus* (32.3%). Fatty acid 16:0 was low in *A. fusca* (9.6%), but high in all Entomobryoidea (23.3%–26.4%), except for *Orchesella villosa* (17.6%). Fatty acid 18:0 was present in trace proportions in *D. sulphureus* (0.9%), but was one of the main FAs in *I. viridis* (15.8%). C20 PUFAs 20:4ω6 and 20:5ω3 were not detected in any of the three Symphypleona species, while 20:5ω3 was high in the Tomoceridae, *Pogonognathellus flavescens* (8.1%), and *Tomocerus vulgaris* (6.1%). Fatty acid 16:1ω7 was highest in *C. denticulata* (8.3%), while 18:1ω7 was highest in the two Tomoceridae, *T. vulgaris* (6.7%) and *P. flavescens* (5.7%). *Pseudosinella immaculata* had a relatively high proportions of FA 14:0 (11.4%).

### 3.2 Phylogenetic signal in FAs of sampled species

**Figure 2:**
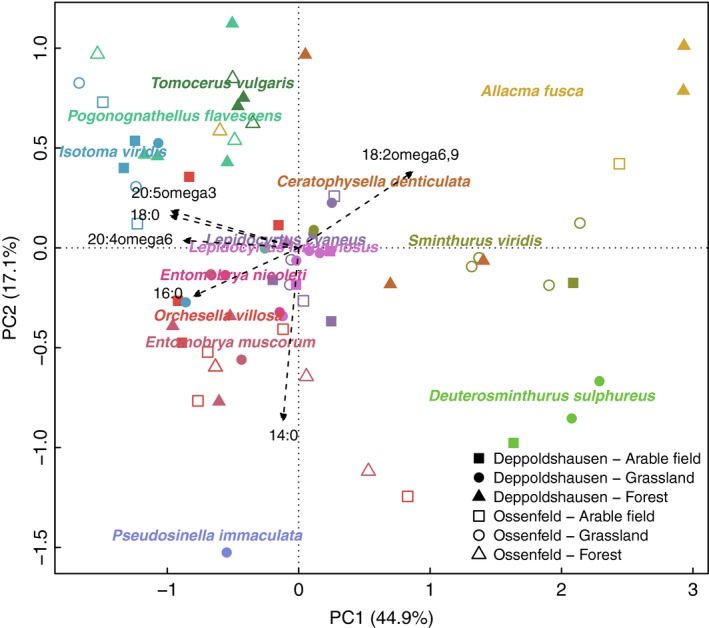
Biplot of principle components analysis using whole fatty acid profiles of 13 field‐sampled Collembola species. Variation explained by each axis is given in parentheses. Position of species name represents its mean score on the axis irrespective of site and habitat. Only fatty acids significantly correlated to the PCs are plotted

**Figure 3:**
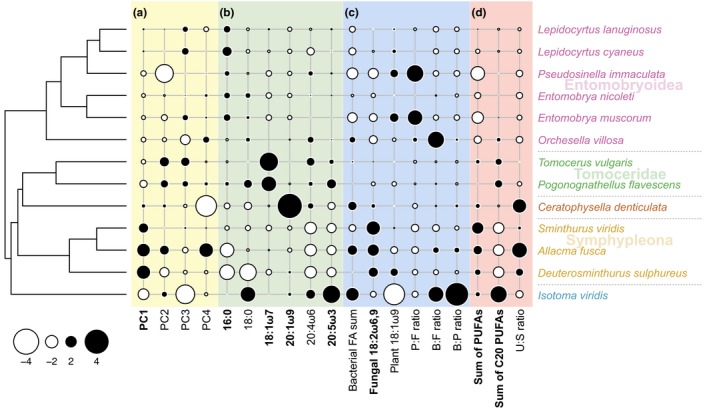
Relationship between phylogeny and selected trait values for field‐sampled Collembola. Trait values were scaled and centralized before plotting. The size of the white and black circles indicates more negative or positive values, respectively. (a) Mean values of the scores of fatty acid profiles on the first four axes in principle components analysis (PCA), (b) proportions of individual fatty acids, (c) proportions of fatty acids derived from bacteria, fungi, or plants, and the ratios between these three, and (d) summed proportions of polyunsaturated fatty acid and C20 polyunsaturated fatty acid, and ratio of unsaturated‐to‐saturated FAs; see Table 2 for abbreviations. Traits exhibiting phylogenetic signal as indicated by both Blomberg's K and Pagel's lambda are marked in bold

The first four PCs together explained 84.2% of the variation in the FA profiles of the Collembola. PC1, representing 44.9% of the variation, showed phylogenetic signal consistent with predictions from the Brownian motion model, as indicated by both Blomberg's K and Pagel's lambda (Table 2). The PCA biplots indicated that three Symphypleona, *A. fusca*,* D. sulphureus,* and *S. viridis*, had higher scores along PC1, which was negatively correlated with FA 18:0, 20:5ω3, 20:4ω6, and 16:0, and positively correlated with FA 18:2ω6,9 (Figures 2 and 3a; Table S4). The remaining PCs, however, exhibited no phylogenetic signal, except PC3 using Blomberg's K without *p*‐value adjustment.

**Table 2:** Phylogenetic signal in fatty acid profiles of 13 field‐collected Collembola species

	Blomberg's K							Pagel's lambda				
	Observed *K*	Permutated *p* (*H*_0_ = 0)		Simulated *p* (*H*_0_ = 1)	Simulated *K* (2.5%–97.5%)			λ	log*L*	log*L*_0_	*p*	
		Unadjusted	BH								Unadjusted	BH
*PCA axis (explained variation)*												
*PC1 (44.9%)	**1.593**	**0.001**	**0.003**	0.121	0.550	–	1.829	**1.278**	**−10.77**	**−18.62**	**<.001**	**<.001**
PC2 (17.0%)	0.694	0.311	0.311					0.262	**−**12.10	**−**12.30	.519	.693
**PC3 (13.1%)**	**0.993**	**0.037**	0.073	0.842	0.543	–	1.817	1.075	**−**9.60	**−**10.61	.155	.309
PC4 (9.1%)	1.059	0.121	0.161					0.000	**−**8.25	**−**8.25	1.000	1.000
*Individual fatty acid*												
8:0	1.696	0.794	0.794					0.000	36.36	36.36	1.000	1.000
10:0	1.854	0.122	0.250					**1.264**	**49.65**	**47.31**	**.031**	.058
**2‐OH 10:0**	7.778	0.075	0.199					**1.278**	**78.54**	**69.63**	**<.001**	**<.001**
**12:0**	4.754	0.118	0.250					**1.278**	**79.56**	**72.41**	**<.001**	**.001**
*14:0*	0.500	0.679	0.721					0.000	27.15	27.15	1.000	1.000
**14:1**	7.580	0.074	0.199					**1.278**	**78.21**	**69.30**	**<.001**	**<.001**
***15:0***	6.775	0.160	0.301					**1.278**	**63.03**	**54.89**	**<.001**	**<.001**
a15:0 (biomarker)	1.476	0.184	0.326					0.000	53.48	53.48	1.000	1.000
i15:0 (biomarker)	1.431	0.546	0.624					0.000	40.02	40.02	1.000	1.000
*16:0	**1.393**	**0.001**	**0.029**	0.241	0.537	–	1.820	**0.973**	**22.90**	**19.31**	**.007**	**.016**
**i16:0** (biomarker)	2.950	0.125	0.250					**1.278**	**76.28**	**71.99**	**.003**	**.009**
16:1ω5	4.840	0.397	0.508					0.000	64.51	64.51	1.000	1.000
***16:1*** **ω** ***7*** (biomarker)	1.564	0.416	0.512					**1.276**	**36.78**	**31.14**	**.001**	**.003**
17:0	2.315	0.698	0.721					0.000	60.91	60.91	1.000	1.000
cy17:0 (biomarker)	3.338	0.277	0.403					0.000	55.60	55.60	1.000	1.000
i17:0 (biomarker)	2.039	0.260	0.396					0.000	33.87	33.87	1.000	1.000
17:1ω8	2.820	0.250	0.396					0.000	53.26	53.26	1.000	1.000
***18:0***	**0.801**	**0.019**	0.070	0.592	0.545	–	1.833	**1.273**	**28.55**	**24.78**	**.006**	**.014**
*18:1 **ω** ***7*** (biomarker)	**1.482**	**0.005**	**0.044**	0.170	0.532	–	1.829	**1.244**	**36.51**	**31.79**	**.002**	**.007**
*18:1*ω*9* (relative biomarker)	**0.920**	**0.020**	0.070	0.968	0.525	–	1.826	1.136	15.54	14.65	.182	.307
*18:2 **ω** ***6,9*** (relative biomarker)	**1.336**	**0.006**	**0.044**	0.279	0.538	–	1.837	**1.165**	**16.04**	**11.88**	**.004**	**.010**
19:0	2.126	0.632	0.698					0.056	55.40	55.39	.909	1.000
cy19:0 (biomarker)	1.521	0.368	0.490					0.000	48.56	48.56	1.000	1.000
*20:1ω9	**3.364**	**0.007**	**0.044**	**<0.001**	**0.547**	–	**1.854**	**1.278**	**60.43**	**51.84**	**<.001**	**<.001**
**20:2ω6,9**	**2.124**	**0.017**	0.070	0.026	0.547	–	1.799	**1.278**	**69.80**	**60.18**	**<.001**	**<.001**
**20:3ω6**	3.530	0.082	0.202					**1.278**	**68.24**	**60.19**	**<.001**	**<.001**
***20:4*** **ω** ***6***	**1.225**	**0.014**	0.070	0.406	0.538	–	1.847	**1.116**	**36.73**	**34.01**	**.020**	**.039**
*20:5 **ω** ***3***	**1.593**	**0.003**	**0.044**	0.109	0.541	–	1.798	**1.229**	**30.54**	**26.02**	**.003**	**.008**
*22:1*ω*9*	4.097	0.201	0.339					**1.171**	**57.28**	**55.06**	**.035**	.062
**22:2**	**3.348**	**0.032**	0.101	<0.001	0.540	–	1.852	**1.278**	**65.05**	**56.89**	**<.001**	**<.001**
*23:0*	4.459	0.343	0.477					0.000	43.74	43.74	1.000	1.000
24:1	0.898	0.460	0.545					0.000	52.84	52.84	1.000	1.000
*FA indices*												
FA number	0.975	0.110	0.135					1.127	**−**26.71	**−**27.62	.177	.277
Sums of bacterial FAs	0.934	0.068	0.107					0.498	25.77	25.17	.273	.334
Plant‐to‐fungal FA ratio (P:F ratio)	0.855	0.299	0.299					0.368	**−**23.84	**−**24.47	.264	.334
Bacterial‐to‐fungal FA ratio (B:F ratio)	1.758	0.097	0.133					0.000	**−**7.22	**−**7.22	1.000	1.000
Bacterial‐to‐plant FA ratio (B:P ratio)	2.471	0.065	0.107					1.050	**−**5.99	**−**7.49	.083	.183
**Unsaturation Index (UI)**	0.659	0.068	0.107					**1.100**	**71.46**	**68.17**	**.010**	**.038**
Sums of saturated FAs (SFAs)	0.788	0.053	0.107					1.110	16.16	14.85	.105	.193
Sums of monounsaturated FAs (MUFAs)	0.933	0.201	0.221					1.253	17.18	16.73	.341	.375
*Sums of polyunsaturated FAs (PUFAs)	**1.128**	**0.012**	**0.044**	0.563	0.535	–	1.763	**0.937**	**19.82**	**16.25**	**.007**	**.038**
*Sums of C20 PUFAs	**1.687**	**0.001**	**0.009**	0.088	0.537	–	1.874	**1.278**	**29.43**	**20.61**	**<.001**	**<.001**
**Unsaturated‐to‐saturated FA ratio (U:S ratio)**	**1.373**	**0.012**	**0.044**	0.245	0.539	–	1.863	**0.898**	**−19.99**	**−22.24**	**.034**	.093

Phylogenetic signal was measured for PCA axes, individual fatty acid proportions and FA indices, and is reported as Blomberg's K combined with permutation significance test (*H*_0_ = 0) and simulation test (*H*_0_ = 1) and as Pagel's lambda with maximum log‐likelihood test. *p*‐values based on permutation testing were corrected using Benjamini & Hochberg’ (BH) method. A significant *K*‐value (*p* < .05) within the 2.5% and 97.5% quantiles of simulated *K* indicates trait evolution as expected under a Brownian motion model. Maximum log likelihood of a trait fit to the given phylogeny (log*L*) was tested against the fit to a lambda transformed phylogeny (log*L*_0_, λ = 0). A significant *p*‐value in Pagel's lambda test indicates phylogenetic signal in that trait. Individual fatty acid differed in proportions between species as indicated by ANOVA (Table S3) is marked in italics. Significant phylogenetic signal of the trait detected by at least one method is marked in bold and by both methods with an asterisk.

Proportions of the FAs 16:0, 18:1ω7, 18:2ω6,9, 20:1ω9, and 20:5ω3 showed significant phylogenetic signal as indicated by Blomberg's K after *p*‐value adjustment. Pagel's lambda further indicated that the FAs 2‐OH 10:0, 12:0, 14:1, 15:0, 16:1ω7, i16:0, 18:0, 20:2ω6,9, 20:3ω6, 20:4ω6, and 22:2 also showed phylogenetic signal after *p*‐value adjustment (Table 2). Phylogenetic signal in FA 16:0 resulted from higher proportions in the clade composed of Lepidocyrtidae and Entomobryidae and lower proportions in *C. denticulata* and Symphypleona. Phylogenetic signal in FA 20:1ω9 resulted from the lack in the clades of Lepidocyrtidae (*Lepidocyrtus* and *Pseudosinella*), Entomobryidae (two *Entomobrya* species), and Sminthuridae (*Allacma* and *Sminthurus*). Notably, the *K*‐value of 20:1ω9 was larger than the 97.5% quantile of simulated *K*‐values, suggesting stronger phylogenetic signal than predicted by the Brownian motion model. Fatty acid 20:5ω3 showed phylogenetic signal due to its consistently lower proportions in *C. denticulata* and Symphypleona, intermediate proportions in Entomobryoidea, higher proportions in Tomoceridae, and even higher proportions in *I. viridis*. Phylogenetic signal in the bacterial biomarker 18:1ω7 reflected higher relative proportion in Tomoceridae and lower in Symphypleona and Entomobryoidea (Table 2, Figure 3b). The fungal biomarker 18:2ω6,9 showed phylogenetic signal, reflecting higher proportions in Symphypleona as well as lower proportions in most Entomobryoidea (Table 2, Figure 3c).

The sum of C20 PUFAs and of all PUFAs exhibited phylogenetic signal according to both Blomberg's K and Pagel's lambda after *p*‐value adjustment. The sum of C20 PUFAs was low in Symphypleona but high in *I. viridis* and the two Tomoceridae species (Table 2, Figure 3d). The sum of all PUFAs, however, was high in Symphypleona, *C. denticulata,* and *I. viridis* but low in Entomobryoidea. The other FA indices, such as ratios between bacterial, fungal, and plant biomarker fatty acids, showed no phylogenetic signal.

### 3.3 Phylogenetic signal in FAs of combined dataset

**Figure 4:**
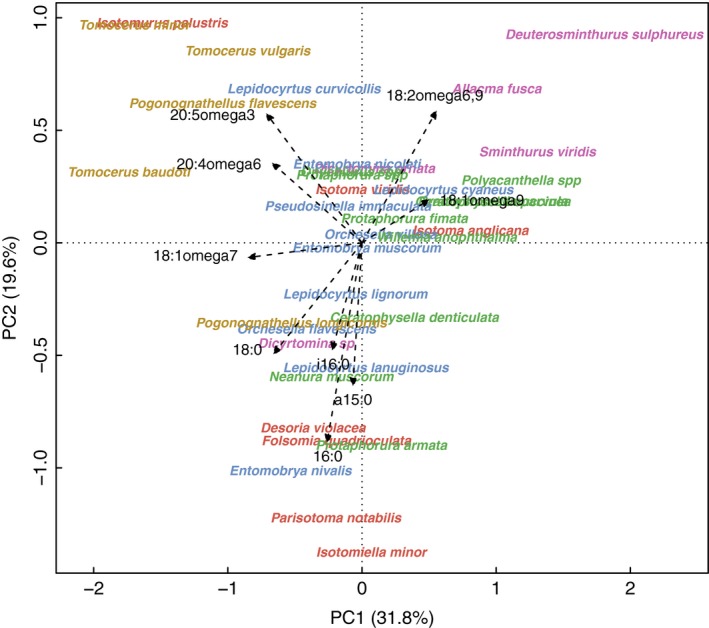
Principle components biplot of the variation in fatty acid profiles of the combined dataset. Variation explained by each axis is given in parentheses. Position of species name represents its mean score on the axis irrespective of reference, site, habitat and treatment; only fatty acids significantly correlated to the PCs are plotted

**Figure 5:**
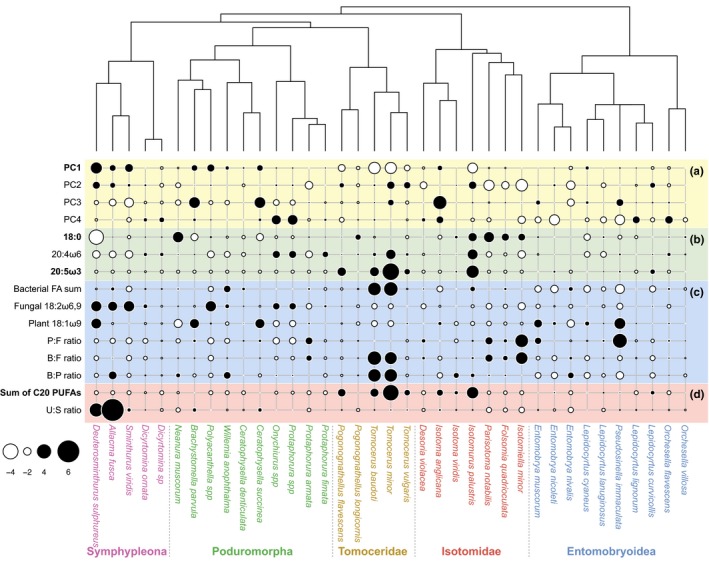
Relationship between phylogeny and selected trait values of Collembola using the combined dataset. Trait values were scaled and centralized before plotting. The size of the white and black circles indicates more negative or positive values, respectively. (a) Mean values of the scores of fatty acid profiles on the first four axes in principle components analysis (PCA), (b) proportions of fatty acids, (c) proportions of fatty acids derived from bacteria, fungi, or plants, as well as the ratios between these three, and (d) summed proportions of C20 polyunsaturated fatty acid and ratio of unsaturated‐to‐saturated FAs; see Table 3 for abbreviations. Traits exhibiting phylogenetic signal as indicated by both Blomberg's K and Pagel's lambda are marked in bold

The first four PCs explained 76.4% of variation in the FA profiles of the 37 species of the combined dataset. PC1 explained 31.8% of the variation in the FA profiles which was positively correlated with FA 18:2ω6,9 and 18:1ω9 and negatively with 18:1ω7, 20:5ω3, 20:4ω6, and 18:0 (Figure 4; Table S4). Phylogenetic signal in species mean scores at PC1 was driven by low scores in Tomoceridae and high scores in the clade of *A. fusca*,* D. sulphureus,* and *S. viridis* (Table 3, Figure 5a). No phylogenetic signal was detected in species mean scores at the other three PCs.

**Table 3:** Phylogenetic signal in fatty acid profiles using the expanded dataset of 37 Collembola species

	Blomberg's K							Pagel's lambda				
	Observed *K*	Permutated *p* (*H*_0_ = 0)		Simulated *p* (*H*_0_ = 1)	Simulated *K* (2.5%–97.5%)			λ	log*L*	log*L*_0_	*p*	
		Unadjusted	BH								Unadjusted	BH
*PCA axis (explained variation)*												
*PC1 (31.4%)	**1.036**	**<0.001**	**<0.001**	0.785	0.598	–	1.698	**0.983**	**−34.91**	**−43.14**	**.000**	**<.001**
PC2 (19.4%)	0.513	0.330	0.440					0.249	**−**33.88	**−**34.15	.461	.615
PC3 (13.3%)	0.541	0.183	0.366					0.013	**−**27.11	**−**27.11	.963	.963
PC4 (11.6%)	0.477	0.444	0.444					0.166	**−**24.04	**−**24.51	.332	.615
*Individual fatty acid*												
a15:0 (biomarker)	0.367	0.903	0.903					0.000	142.10	142.10	1.000	1.000
*i15:0* (biomarker)	0.973	0.379	0.587					0.023	119.77	119.77	.964	1.000
*16:0*	0.533	0.293	0.587					0.189	45.67	45.45	.515	.735
i16:0 (biomarker)	0.433	0.719	0.885					0.000	147.66	147.66	1.000	1.000
*16:1*ω*7* (biomarker)	0.551	0.329	0.587					0.356	90.66	89.16	.084	.209
i17:0 (biomarker)	0.859	0.854	0.903					0.000	134.92	134.92	1.000	1.000
*18:0	**0.742**	**0.002**	**0.022**	0.338	0.591	–	1.664	**1.086**	**75.62**	**71.19**	**.003**	**.015**
18:1ω7 (biomarker)	**0.661**	**0.025**	0.125	0.209	0.605	–	1.686	**0.638**	**78.38**	**75.82**	**.024**	.095
*18:1*ω*9* (relative biomarker)	0.480	0.432	0.587					0.000	46.33	46.33	1.000	1.000
***18:2*** **ω** ***6,9*** (relative biomarker)	0.683	0.130	0.417					**0.619**	**43.22**	**40.00**	**.011**	**.037**
cy19:0 (biomarker)	1.180	0.373	0.587					0.000	120.35	120.35	1.000	1.000
*20:1*ω*9*	0.608	0.440	0.587					0.219	104.49	104.26	.498	.735
20:2ω6,9	3.473	0.328	0.587					0.026	171.24	171.23	.870	1.000
*20:3*ω*6*	2.916	0.840	0.903					0.044	107.74	107.73	.912	1.000
*20:4*ω*6*	**0.644**	**0.031**	0.125	0.104	0.599	–	1.711	0.492	89.31	89.03	.454	.735
*20:5 **ω** ***3***	**0.853**	**0.003**	**0.022**	0.641	0.601	–	1.678	**0.820**	**78.16**	**73.26**	**.002**	**.015**
*FA indices*												
Sum of bacterial FAs	0.524	0.296	0.453					0.213	70.39	69.60	.208	.498
Plant‐to‐fungal FA ratio (P:F ratio)	0.584	0.370	0.453					0.187	**−**60.64	**−**61.02	.381	.544
Bacterial‐to‐fungal FA ratio (B:F ratio)	0.620	0.228	0.453					0.370	**−**25.67	**−**26.61	.169	.498
Bacterial‐to‐plant FA ratio (B:P ratio)	0.518	0.452	0.453					0.102	11.25	11.02	.498	.553
Unsaturation Index (UI)	0.565	0.090	0.448					0.358	170.89	170.43	.337	.544
Sum of saturated FAs (SFAs)	0.569	0.183	0.453					0.270	33.43	33.16	.468	.553
Sum of monounsaturated FAs (MUFAs)	0.486	0.453	0.453					0.000	46.46	46.46	1.000	1.000
Sum of polyunsaturated FAs (PUFAs)	0.529	0.340	0.453					0.273	40.21	39.55	.249	.498
Sum of C20 PUFAs*	**0.811**	**0.003**	**0.034**	0.525	0.603	–	1.642	**0.803**	**76.98**	**72.96**	**.005**	**.046**
Unsaturated‐to‐saturated FA ratio (U:S ratio)	1.166	0.148	0.453					**0.698**	**−60.38**	**−63.37**	**.014**	.072

Phylogenetic signal was measured for PCA axes, individual fatty acid proportions and FA indices, and is reported as Blomberg's K combined with permutation significance test (*H*_0_ = 0) and simulation test (*H*_0_ = 1) and as Pagel's lambda with maximum log‐likelihood test. *p*‐values based on permutation testing were corrected using Benjamini & Hochberg’ (BH) method. A significant *K*‐value (*p* < .05) within the 2.5% and 97.5% quantiles of simulated *K* indicates trait evolution as expected under a Brownian motion model. Maximum log likelihood of a trait fit to the given phylogeny (log*L*) was tested against the fit to a lambda transformed phylogeny (log*L*_0_, λ = 0). A significant *p*‐value in Pagel's lambda test indicates phylogenetic signal in that trait. Individual fatty acid differed in proportions between species as indicated by ANOVA (Table S5) is marked in italic. Significant phylogenetic signal of the trait detected by at least one method is marked in bold and by both methods with an asterisk.

Analyses of phylogenetic signal in the mean proportions of individual FAs (Table S5) suggested that FA 18:0 and 20:5ω3 exhibited phylogenetic signal as indicated by both Blomberg's K and Pagel's lambda (Table 3). Fatty acid 18:0 was high in the clade composed of *Isotomurus palustris*,* Parisotoma notabilis*,* Isotomiella minor,* and *Folsomia quadrioculata*, while FA 20:5ω3 was mainly present in Tomoceridae but absent in Poduromorpha (Figure 5b). The fungal biomarker 18:2ω6,9 exhibited phylogenetic signal only by Pagel's lambda; however, plant biomarker 18:1ω9, the sum of bacterial FAs, and the ratios between fungal, plant, and bacterial FAs showed no phylogenetic signal (Table 3, Figure 5c). The sum of C20 PUFAs showed phylogenetic signal as indicated by both Blomberg's K and Pagel's lambda (Table 3). It was high in *Tomocerus*, intermediate in Entomobryoidea, and low in the clade of *I. minor*,* F. quadrioculata,* and *P. notabilis*, as well as the clade of *S. viridis*,* A. fusca,* and *D. sulphureus* (Figure 5d).

## 4 DISCUSSION

Studies on FAs in Collembola usually have used one or two species in laboratory cultures under different conditions (Chamberlain, Bull, Black, Ineson, & Evershed, 2005; Haubert et al., 2008; van Dooremalen & Ellers, 2010) or analyzed FAs of field‐sampled species but with limited numbers of species sampled from one habitat type, that is, forest (Chamberlain & Black, 2005; Ruess et al., 2007; Ferlian et al., 2015) or arable fields (Haubert et al., 2009; Ngosong, Raupp, Scheu, & Ruess, 2009; Sechi et al., 2014). This study is the first to measure phylogenetic signal in FA compositions of field‐sampled Collembola from different habitats using a phylogenetic comparative method. Our results suggest that although habitat effects on FA profiles were minor, FA compositions differed significantly between species and generally displayed phylogenetic signal, as indicated by the first PC axis for both field‐sampled and combined datasets.

### 4.1 Fatty acids, animal physiology, and phylogenetics (β niche traits)

Phylogenetic signal was detected in C20 PUFAs and proportions of 20:5ω3 in both our field‐sampled and combined datasets, supporting the first hypothesis that closely related Collembola species have similar proportions of C20 PUFAs. In field‐sampled Collembola, Symphypleona contained lower proportions of C20 PUFAs than Entomobryomorpha, consistent with previous findings (Chamberlain & Black, 2005). Collembola may have the ability to synthesize C20 PUFAs from precursors, as indicated by laboratory experiments in which a high proportion of C20 PUFAs was found in Isotomidae and Onychiuridae fed with food containing no PUFAs (Chamberlain & Black, 2005). In insects, C20 PUFAs are essential for biosynthesis of prostaglandins and eicosanoids, which are important for reproduction and immune response, and related to temperature and humidity of the habitat (Stanley‐Samuelson, Dell, & Ogg, 1992; Stanley‐Samuelson, 1994). Accordingly, the phylogenetic signal of C20 PUFA in different Collembola lineages presumably reflects an evolutionary constraint of physiological functions related to these FAs. Symphypleona predominantly live at the soil surface where humidity fluctuates with some dry periods, while the other taxa, such as Isotomidae, Tomoceridae, and Poduromorpha, predominantly dwell in soil where humidity is high and relatively stable. Physiological constraints on the proportions of C20 PUFAs within phylogenetic lineages likely reflect the different soil horizons the species live in. However, the linkage between C20 PUFAs and the adaptation of species to different soil layers requires further examination of the functions of C20 PUFAs in Collembola.

### 4.2 Fatty acids, food resources, and phylogenetics (α niche traits)

Among biomarker FAs, only three markers (18:1ω7, 18:2ω6,9 and 20:1ω9) exhibited phylogenetic signal in the field‐sampled dataset, while the combined dataset showed phylogenetic signal in PC1 that correlated with 18:1ω7 and two other biomarker FAs (18:1ω9 and 18:2ω6,9). Fatty acid 18:1ω7 is an absolute bacterial biomarker synthesized exclusively by bacteria (Ruess & Chamberlain, 2010; Ferlian et al., 2015). High proportions of 18:1ω7 in Tomoceridae of our field‐sampled dataset indicate that they fed heavily on bacteria at the study sites, whereas Entomobryoidea and Symphypleona consumed less food resources containing this FA. Presumably, feeding on bacteria has been restricted to certain Collembola phylogenetic groups during evolutionary history, but this hypothesis needs further testing.

Phylogenetic signal was detected in the proportion of 18:2ω6,9 in the field‐sampled dataset. Fatty acid 18:2ω6,9 was higher in Symphypleona, consistent with findings of Chamberlain and Black (2005) where two Symphypleona species also had higher proportions of it than the other species sampled from a deciduous woodland. High proportions of 18:2ω6,9 are found in body tissue under a fungus‐based diet and thus have been used as indicator of fungal food resources (Ruess & Chamberlain, 2010; Ferlian et al., 2015). However, 18:2ω6,9 can be synthesized by higher insects (Cripps, Blomquist, & de Renobales, 1986) and therefore may also be related to species’ physiology. Several groups of Collembola are able to synthesize 18:2ω6,9, including Isotomidae, Poduromorpha, and Entomobryoidea (Chamberlain et al., 2004; Chamberlain & Black, 2005; Haubert, Häggblom, Langel, Scheu, & Ruess, 2006), but this has not been tested for Symphypleona. Nevertheless, high proportions of 18:2ω6,9 still may reflect a fungal‐based diet in Symphypleona (Ruess et al., 2005; Ruess & Chamberlain, 2010), but biosynthesis must be excluded by laboratory experiments before concluding that there is an evolutionary constraint in fungal feeding among different Collembola phylogenetic groups.

Strong phylogenetic signal was detected in the proportion of 20:1ω9 of the field‐sampled dataset. Collembola unlikely are able to biosynthesize 20:1ω9 *de novo* but rather incorporate it from food, presumably from nematodes (Ruess, Häggblom, Langel, & Scheu, 2004; Ruess et al., 2005). The lack of 20:1ω9 in the clade of Lepidocyrtidae and Entomobryidae and the clade of Sminthuridae indicates that at our study sites, these Collembola did not feed on nematodes, while the remaining species, especially *C. denticulate*, may have consumed nematodes. However, when more species and measurements were included from other studies (the combined dataset), no phylogenetic signal was found in proportion of 20:1ω9, nor in site scores on PC3 and PC4 which were correlated with 20:1ω9. Phylogenetic signal found in our field‐sampled dataset may therefore be an exception. Indeed, Collembola from different forest sites have been shown with different proportions of 20:1ω9, presumably related to the amounts of resources in the environment (Ruess et al., 2005).

The ratios of bacterial, fungal, and plant FAs, which have been used to assign species to feeding guilds, did not show phylogenetic signal. These results partially support our second hypothesis that food resource FAs are a phylogenetically independent trait, implying niche partitioning in food resources among closely related species, thereby favoring species coexistence. Phylogenetic signal may be reduced due to a mixture of convergent evolution and conservatism in traits, or a developed trait irrespective of species’ evolution (i.e., a phylogenetically random trait). Our analyses used ratio as a continuous variable, and the ability to detect phylogenetic signal may be reduced due to large intraspecific variation or measurement errors (Ives et al., 2007). Indeed, Collembola are described as generalists able to consume a broad spectrum of food resources, exhibiting a considerable intraspecific variation in biomarker FA proportions from laboratory experiments (Chamberlain et al., 2005; Ruess et al., 2005; Haubert, Pollierer, & Scheu, 2011). In field samples, the variation is expected to be even larger, and it is possible that consumption of food resources is influenced by other co‐occurring species.

Fatty acid composition complements stable isotopes in analyzing the trophic niche of soil biota (Ferlian et al., 2015). Using taxonomy as a surrogate of phylogenetic relationships with stable isotope data suggests conservatism in Collembola trophic niches (Potapov, Semenina, Korotkevich, Kuznetsova, & Tiunov, 2016), in contrast to the findings of the current study. Thus, Collembola feeding traits are, on one hand, likely to have been constrained along species’ evolutionary history; on the other hand, they may retain variability to reduce competition. More data on trophic niches and food resources of Collembola species from different phylogenetic groups are necessary to test this hypothesis.

### 4.3 Traits and species coexistence in soil

Species can coexist when they have similar β niche traits and different α niche traits (Silvertown et al., 2006). Phylogenetic signal detected in C20 PUFAs (β niche) but general lability in biomarker FAs and bacterial, fungal, and plant FA ratios (α niche) may explain how different Collembola species coexist. Moreover, explicitly testing phylogenetic conservatism in functional traits is crucial for community phylogenetic and trait‐based approaches, because the traits are mechanistic links by which phylogenetic history can influence contemporary ecological processes in communities (Cavender‐Bares, Kozak, Fine, & Kembel, 2009). Phylogenetic signal measurement in this study therefore, represents a starting point to further investigate evolutionary hypotheses on the adaptation of soil animals to environmental conditions (Revell, Harmon, & Collar, 2008; Cooper, Jetz, & Freckleton, 2010), thereby linking community phylogenetic and trait‐based approaches with coexistence studies on soil biota.

## 5 CONCLUSIONS

Our results show that Collembola FA profiles generally exhibit phylogenetic signal. We found phylogenetic signal in C20 PUFA proportions of Collembola, while biomarker FAs differed among species but were generally labile. These patterns suggest that (1) physiological properties of species may be constrained during evolutionary history, resulting in phylogenetically related species having similar physiologically related FAs, and (2) Collembola food resources are phylogenetically labile, favoring species coexistence. Our study is the first to report phylogenetic signal in the fatty acid compositions of animals in the context of species coexistence. The results form a starting point to further investigate evolutionary hypotheses on the adaptation of soil animals to environmental conditions. Integrating phylogenetic comparative methods and community phylogenetic and trait‐based approaches may help identify evolutionary and ecological forces driving and maintaining communities in soil.

## CONFLICT OF INTEREST

None declared.

## AUTHOR CONTRIBUTIONS

TWC and SS conceived and designed the study; TWC and PS performed the study; TWC, IS, and SS wrote the manuscript. All authors revised and approved the manuscript.

## DATA ACCESSIBILITY

Concatenated alignment and phylogenetic trees generated from this study were deposited in TreeBASE http://purl.org/phylo/treebase/phylows/study/TB2:S20409).
