# Supplementary figures and images for: Neutral lipid fatty acid composition as trait and constraint in Collembola evolution
Source: Ecol Evol. 2017 Oct 16;7(22):9624–38. doi: 10.1002/ece3.3472 (PMC5696395; doi:10.1002/ece3.3472)

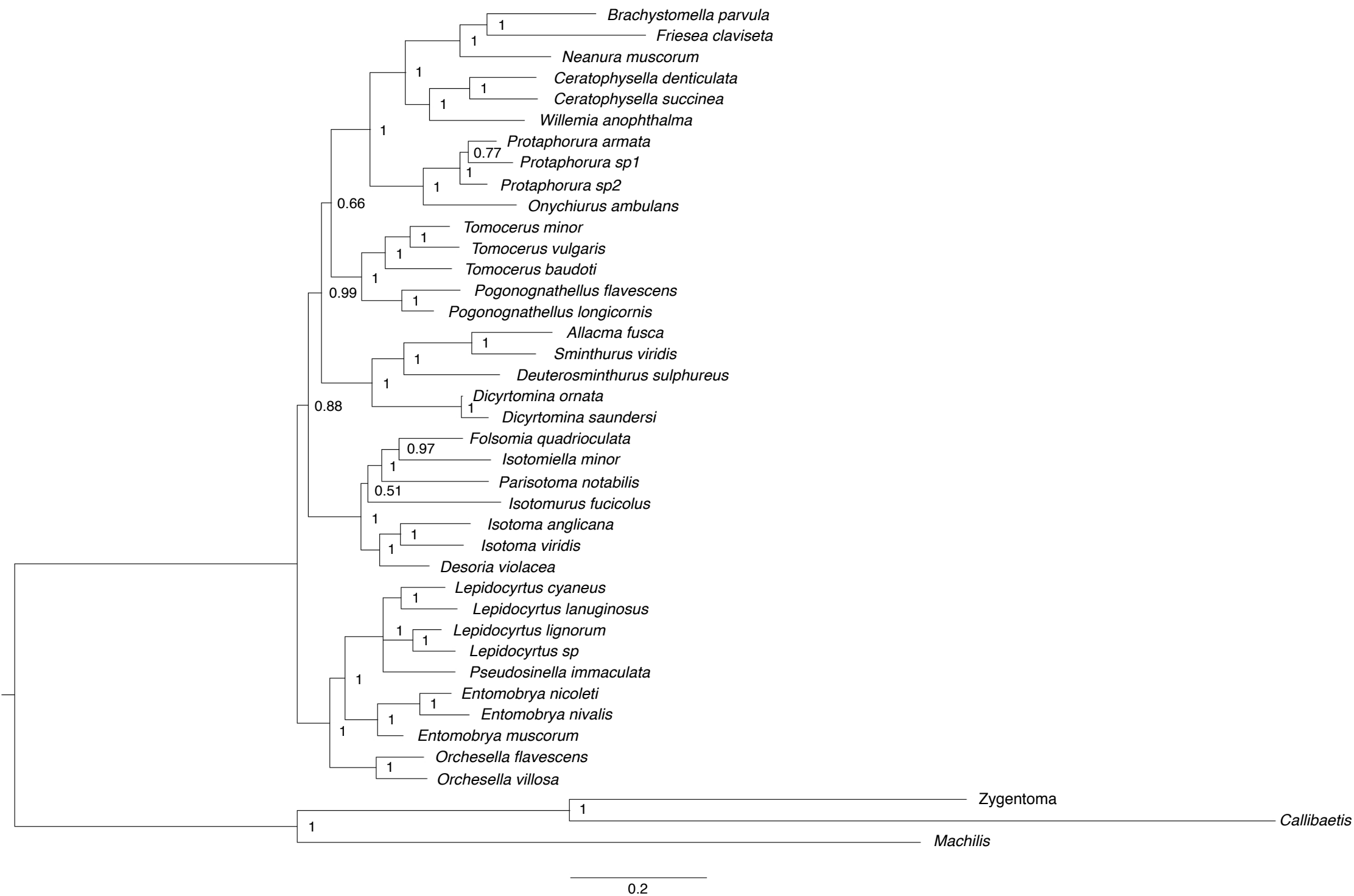

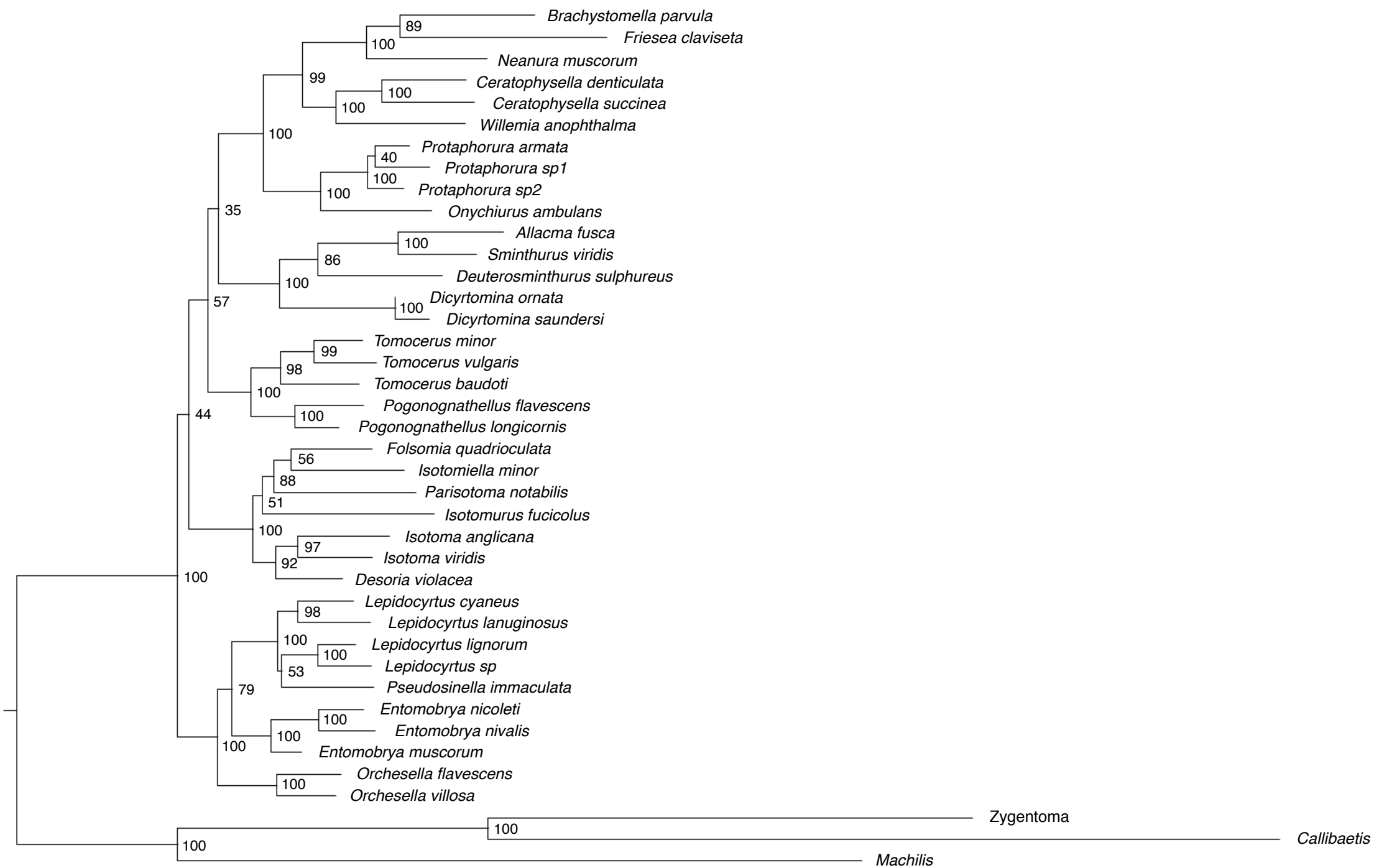

Figure S2

Supplement: Supplementary file 1 [file ECE3-7-9624-s001.pdf]
