# Supplementary material for: Neutral lipid fatty acid composition as trait and constraint in Collembola evolution
Source: Ecol Evol. 2017 Oct 16;7(22):9624–38. doi: 10.1002/ece3.3472 (PMC5696395; doi:10.1002/ece3.3472)
Supplement: Supplementary file 2 [file ECE3-7-9624-s002.docx]

| **Table S1** |  |  |  |  |  |  |
| --- | --- | --- | --- | --- | --- | --- |
| NCBI accession numbers of sequences used to construct the molecular phylogeny of Collembola. | | | | | | |
| Species | Molecular markers | | | | | |
|  | 18S rRNA | 28S rRNA | | | Histone 3 | COI |
|  |  | D1 | D2 | D3-D5 |  |  |
| Zygentoma (outgroup) | AF370791 | AY859557 | AY859557 | AY859557 | AY749703 | KM535783 |
| *Callibaetis* (outgroup) | AY521826 | AY521735 | AY521735 | AY521735 | AY521695 | JF735107 |
| *Machilis* (outgroup) | AY210811 | AY210810 | AY210810 | AY210810 | AY338644 | JN970940 |
| *Allacma fusca* | KY230702 | KY230832 | KY230832 | KY230935 |  | KY231097 |
| *Brachystomella parvula* | KY230724 | KY230822 | KY230822 | KY230925 | KY231066 | KY231088 |
| *Ceratophysella denticulata* | KY230747 | KY230847 | KY230847 | KY230948 | KY231036 | KY231107 |
| *Ceratophysella succinea* |  | KY230885 | KY230885 | KY230990 | KY231065 | KY231136 |
| *Desoria violacea* | KY230736 | KY230833 | KY230833 | KY230902 | KY231003 | KY231098 |
| *Deuterosminthurus sulphureus* | KY230753 | KY230830 | KY230830 | KY230933 | KY231024 | KY231095 |
| *Dicyrtomina ornata* | KY230768 | KY230840 | KY230840 | KY230968 | KY231046 | KY231125 |
| *Dicyrtomina saundersi* | EU368611 | EF199974 | EF199974 | EF199974 |  |  |
| *Entomobrya muscorum* | KY230710 | KY230806 | KY230806 | KY230909 | KY231010 |  |
| *Entomobrya nicoleti* | KY230740 | KY230838 | KY230838 | KY230941 | KY231030 | KY231119 |
| *Entomobrya nivalis* |  | LK024313 | LK024313 |  |  | HG422598 |
| *Folsomia quadrioculata* | KY230755 | KY230853 | KY230853 | KY230955 | KF684772 | KF684607 |
| *Friesea claviseta* | KY230727 | KY230826 | KY230826 | KY230929 | KY231020 | KY231092 |
| *Isotoma anglicana* | KY230779 | KY230873 | KY230873 | KY230975 | KY231053 | KY231076 |
| *Isotoma viridis* | KY230708 | KY230835 | KY230835 | KY230938 | KY231028 | KY231129 |
| *Isotomiella minor* | KY230744 | KY230843 | KY230843 | KY230945 | KY231034 | KY231103 |
| *Isotomurus fucicolus* | KY230704 | KY230834 | KY230834 | KY230937 | KY231057 | KY231099 |
| *Lepidocyrtus cyaneus* | KY230751 | KY230851 | KY230851 | KY230952 | KY231040 | KY231111 |
| *Lepidocyrtus lanuginosus* | KY230748 | KY230848 | KY230848 | KY230949 | KY231037 | KY231108 |
| *Lepidocyrtus lignorum* | KY230749 | KY230849 | KY230849 | KY230950 | KY231038 | KY231109 |
| *Lepidocyrtus sp* | KY230750 | KY230850 | KY230850 | KY230951 | KY231039 | KY231110 |
| *Neanura muscorum* | AY555520 | AJ251733 | AJ251733 | AJ251733 |  | AY555544 |
| *Onychiurus ambulans* | AY555518 | AF483384 | AF483442 | HQ731961 | AY555564 | HQ732075 |
| *Orchesella flavescens* | KY230714 | KY230811 | KY230811 | KY230997 | KY231015 | KY231082 |
| *Orchesella villosa* | KY230715 | KY230812 | KY230812 | KY230904 | KY231005 | KY231091 |
| *Parisotoma notabilis* | KY230772 | KY230872 | KY230872 | KY230974 | KY231052 | KY231128 |
| *Pogonognathellus flavescens* | KY230717 | KY230814 | KY230814 | KY230917 | KY231016 | KY231083 |
| *Pogonognathellus longicornis* |  | KY230845 | KY230845 | KY230946 | KY231035 | KY231105 |
| *Protaphorura armata* |  | AF483391 | AF483449 | HQ731965 |  | HQ732078 |
| *Protaphorura sp1* | KY230789 | KY230884 | KY230884 | KY230989 | KY231063 | HG422585 |
| *Protaphorura sp2* | KY230719 | KY230856 | KY230856 | KY230959 | KY231064 | KY231116 |
| *Pseudosinella immaculata* | KY230712 |  |  | KY230911 | KY231012 | KY231078 |
| *Sminthurus viridis* | KY230701 | KY230880 | KY230880 | KY230983 | KY231001 | JN970939 |
| *Tomocerus baudoti* |  |  | JX261697 |  |  | JX261845 |
| *Tomocerus minor* | AY555516 | AF483406 | JX261700 | HQ731971 | AY555562 | HM398041 |
| *Tomocerus vulgaris* | KY230777 | KY230815 | KY230815 | KY230918 | KY231058 | KY231131 |
| *Willemia anophthalma* | KY230726 | KY230869 | KY230869 | KY230927 |  | KY231090 |

| **Table S2** |  |  |  |  |  |  |  |  |  |  |  |  |  |  |  |  |  |  |  |  |  |  |  |  |
| --- | --- | --- | --- | --- | --- | --- | --- | --- | --- | --- | --- | --- | --- | --- | --- | --- | --- | --- | --- | --- | --- | --- | --- | --- |
| NLFAs proportions (mean ± standard error, %) for the 13 Collembola species collected in this study, pooled for sites and habitats. Fatty acid names in bold indicate significant difference between different species on fatty acid proportions in ANOVA (Table S3). Different letters next to fatty acid proportions indicate significant differences (*P* < 0.05) between species in Tukey's HSD test. | | | | | | | | | | | | | | | | | | | | | | | | |
| Fatty acid | *Lepidocyrtus lanuginosus* | | | | *Lepidocyrtus cyaneus* | | | | *Pseudosinella immaculata* | | | | *Entomobrya nicoleti* | | | | *Entomobrya muscorum* | | | | *Orchesella villosa* | | | |
| 8:0 | 0.0 | ± | 0.0 |  | 1.8 | ± | 0.7 |  | 0.0 | |  |  | 0.0 | ± | 0.0 |  | 1.2 | ± | 0.9 |  | 3.5 | ± | 1.2 |  |
| 10:0 | 0.0 | ± | 0.0 |  | 0.1 | ± | 0.1 |  | 0.0 | |  |  | 1.1 | ± | 1.1 |  | 0.3 | ± | 0.3 |  | 1.9 | ± | 0.8 |  |
| 2-OH 10:0 | 0.0 | ± | 0.0 |  | 0.0 | ± | 0.0 |  | 0.0 | |  |  | 0.0 | ± | 0.0 |  | 0.0 | ± | 0.0 |  | 0.0 | ± | 0.0 |  |
| 12:0 | 0.0 | ± | 0.0 |  | 0.0 | ± | 0.0 |  | 0.0 | |  |  | 0.0 | ± | 0.0 |  | 0.0 | ± | 0.0 |  | 0.3 | ± | 0.3 |  |
| **14:0** | **1.6** | **±** | **0.3** | **abc** | **0.6** | **±** | **0.2** | **b** | **11.4** | | **d** |  | **1.4** | **±** | **0.1** | **abcd** | **1.7** | **±** | **0.6** | **abc** | **5.0** | **±** | **1.2** | **cd** |
| 14:1 | 0.0 | ± | 0.0 |  | 0.0 | ± | 0.0 |  | 0.0 | |  |  | 0.0 | ± | 0.0 |  | 0.0 | ± | 0.0 |  | 0.0 | ± | 0.0 |  |
| **15:0** | **0.0** | **±** | **0.0** | **b** | **0.0** | **±** | **0.0** | **b** | **0.0** | | **b** |  | **0.0** | **±** | **0.0** | **b** | **0.0** | **±** | **0.0** | **b** | **0.0** | **±** | **0.0** | **b** |
| a15:0 | 0.0 | ± | 0.0 |  | 0.0 | ± | 0.0 |  | 0.0 | |  |  | 0.9 | ± | 0.9 |  | 0.0 | ± | 0.0 |  | 0.9 | ± | 0.5 |  |
| i15:0 | 0.0 | ± | 0.0 |  | 0.0 | ± | 0.0 |  | 0.0 | |  |  | 0.7 | ± | 0.7 |  | 0.0 | ± | 0.0 |  | 2.7 | ± | 1.0 |  |
| **16:0** | **24.6** | **±** | **0.6** | **c** | **26.4** | **±** | **0.7** | **c** | **23.3** | | **abc** |  | **24.0** | **±** | **0.4** | **bc** | **24.6** | **±** | **1.1** | **c** | **17.6** | **±** | **2.5** | **bc** |
| 16:1ω5 | 0.0 | ± | 0.0 |  | 0.0 | ± | 0.0 |  | 0.0 | |  |  | 0.6 | ± | 0.6 |  | 0.0 | ± | 0.0 |  | 0.2 | ± | 0.2 |  |
| **16:1ω7** | **1.9** | **±** | **0.1** | **bc** | **2.0** | **±** | **0.6** | **b** | **1.2** | | **abc** |  | **0.8** | **±** | **0.8** | **bc** | **1.4** | **±** | **0.4** | **b** | **2.3** | **±** | **0.6** | **bc** |
| i16:0 | 0.0 | ± | 0.0 |  | 0.0 | ± | 0.0 |  | 0.0 | |  |  | 0.0 | ± | 0.0 |  | 0.2 | ± | 0.2 |  | 0.2 | ± | 0.2 |  |
| 17:0 | 0.2 | ± | 0.2 |  | 0.0 | ± | 0.0 |  | 0.0 | |  |  | 0.0 | ± | 0.0 |  | 0.0 | ± | 0.0 |  | 0.1 | ± | 0.1 |  |
| 17:1ω8 | 0.0 | ± | 0.0 |  | 0.0 | ± | 0.0 |  | 0.0 | |  |  | 1.0 | ± | 1.0 |  | 0.0 | ± | 0.0 |  | 0.2 | ± | 0.2 |  |
| i17:0 | 0.0 | ± | 0.0 |  | 0.0 | ± | 0.0 |  | 0.0 | |  |  | 0.6 | ± | 0.6 |  | 0.3 | ± | 0.3 |  | 1.0 | ± | 0.6 |  |
| cy17:0 | 0.0 | ± | 0.0 |  | 0.0 | ± | 0.0 |  | 0.0 | |  |  | 0.0 | ± | 0.0 |  | 0.0 | ± | 0.0 |  | 0.3 | ± | 0.2 |  |
| **18:0** | **7.3** | **±** | **0.8** | **abc** | **6.9** | **±** | **0.9** | **abc** | **7.7** | | **abc** |  | **11.2** | **±** | **1.8** | **abc** | **10.4** | **±** | **1.7** | **bc** | **10.8** | **±** | **1.9** | **bc** |
| **18:1ω7** | **1.2** | **±** | **0.2** | **a** | **0.8** | **±** | **0.4** | **a** | **0.0** | | **abc** |  | **0.0** | **±** | **0.0** | **a** | **0.0** | **±** | **0.0** | **a** | **1.5** | **±** | **0.8** | **a** |
| **18:1ω9** | **36.3** | **±** | **1.8** | **a** | **38.0** | **±** | **1.6** | **a** | **42.2** | | **ab** |  | **34.6** | **±** | **6.1** | **ab** | **43.1** | **±** | **4.4** | **a** | **29.9** | **±** | **3.5** | **a** |
| **18:2ω6,9** | **20.9** | **±** | **1.5** | **abc** | **17.1** | **±** | **1.7** | **acd** | **7.0** | | **acd** |  | **14.2** | **±** | **2.1** | **abcd** | **9.6** | **±** | **1.4** | **d** | **9.6** | **±** | **1.4** | **d** |
| 19:0 | 0.0 | ± | 0.0 |  | 0.3 | ± | 0.2 |  | 0.0 | |  |  | 0.0 | ± | 0.0 |  | 0.0 | ± | 0.0 |  | 0.4 | ± | 0.4 |  |
| cy19:0 | 0.4 | ± | 0.3 |  | 0.1 | ± | 0.1 |  | 0.0 | |  |  | 1.3 | ± | 1.3 |  | 0.0 | ± | 0.0 |  | 0.0 | ± | 0.0 |  |
| 20:1ω9 | 0.0 | ± | 0.0 |  | 0.0 | ± | 0.0 |  | 0.0 | |  |  | 0.0 | ± | 0.0 |  | 0.0 | ± | 0.0 |  | 0.2 | ± | 0.2 |  |
| 20:2ω6,9 | 0.0 | ± | 0.0 |  | 0.0 | ± | 0.0 |  | 0.0 | |  |  | 0.0 | ± | 0.0 |  | 0.0 | ± | 0.0 |  | 0.1 | ± | 0.1 |  |
| 20:3ω6 | 0.0 | ± | 0.0 |  | 0.0 | ± | 0.0 |  | 0.0 | |  |  | 0.0 | ± | 0.0 |  | 0.0 | ± | 0.0 |  | 0.5 | ± | 0.4 |  |
| **20:4ω6** | **2.1** | **±** | **0.2** | **abd** | **1.0** | **±** | **0.3** | **bcd** | **3.9** | | **abcd** |  | **3.1** | **±** | **0.1** | **abcd** | **2.7** | **±** | **0.6** | **ad** | **4.2** | **±** | **0.7** | **a** |
| **20:5ω3** | **3.4** | **±** | **0.2** | **cd** | **4.6** | **±** | **0.4** | **de** | **3.4** | | **abcdef** |  | **3.7** | **±** | **0.4** | **bcdef** | **3.9** | **±** | **0.9** | **cde** | **4.9** | **±** | **1.1** | **de** |
| **22:1ω9** | **0.2** | **±** | **0.2** | **ab** | **0.0** | **±** | **0.0** | **a** | **0.0** | | **ab** |  | **0.0** | **±** | **0.0** | **ab** | **0.0** | **±** | **0.0** | **a** | **0.0** | **±** | **0.0** | **a** |
| 22:2 | 0.0 | ± | 0.0 |  | 0.0 | ± | 0.0 |  | 0.0 | |  |  | 0.0 | ± | 0.0 |  | 0.0 | ± | 0.0 |  | 0.7 | ± | 0.7 |  |
| **23:0** | **0.0** | **±** | **0.0** | **a** | **0.0** | **±** | **0.0** | **a** | **0.0** | | **ab** |  | **0.5** | **±** | **0.5** | **ab** | **0.2** | **±** | **0.2** | **a** | **0.4** | **±** | **0.2** | **a** |
| 24:1 | 0.0 | ± | 0.0 |  | 0.1 | ± | 0.1 |  | 0.0 | |  |  | 0.0 | ± | 0.0 |  | 0.3 | ± | 0.3 |  | 0.5 | ± | 0.3 |  |

| **Table S2** |  |  |  |  |  |  |  |  |  |  |  |  |  |  |  |  |  |  |  |  |  |  |  |  |  |  |  |  |
| --- | --- | --- | --- | --- | --- | --- | --- | --- | --- | --- | --- | --- | --- | --- | --- | --- | --- | --- | --- | --- | --- | --- | --- | --- | --- | --- | --- | --- |
| (Continued) |  |  |  |  |  |  |  |  |  |  |  |  |  |  |  |  |  |  |  |  |  |  |  |  |  |  |  |  |
| Fatty acid | *Tomocerus vulgaris* | | | | *Pogonognathellus flavescens* | | | | *Sminthurus viridis* | | | | *Allacma fusca* | | | | *Deuterosminthurus sulphureus* | | | | *Ceratophysella denticulata* | | | | *Isotoma viridis* | | | |
| 8:0 | 0.0 | ± | 0.0 |  | 0.9 | ± | 0.7 |  | 1.2 | ± | 1.2 |  | 4.9 | ± | 1.7 |  | 1.7 | ± | 1.7 |  | 0.0 | ± | 0.0 |  | 0.0 | ± | 0.0 |  |
| 10:0 | 0.0 | ± | 0.0 |  | 0.5 | ± | 0.3 |  | 0.0 | ± | 0.0 |  | 0.0 | ± | 0.0 |  | 0.0 | ± | 0.0 |  | 0.0 | ± | 0.0 |  | 1.6 | ± | 0.7 |  |
| 2-OH 10:0 | 0.0 | ± | 0.0 |  | 0.0 | ± | 0.0 |  | 0.0 | ± | 0.0 |  | 0.0 | ± | 0.0 |  | 0.0 | ± | 0.0 |  | 0.0 | ± | 0.0 |  | 0.4 | ± | 0.3 |  |
| 12:0 | 0.0 | ± | 0.0 |  | 0.0 | ± | 0.0 |  | 0.0 | ± | 0.0 |  | 0.0 | ± | 0.0 |  | 0.0 | ± | 0.0 |  | 0.0 | ± | 0.0 |  | 0.2 | ± | 0.2 |  |
| **14:0** | **0.3** | **±** | **0.3** | **ab** | **1.0** | **±** | **0.8** | **ab** | **1.5** | **±** | **0.3** | **abc** | **0.4** | **±** | **0.4** | **ab** | **5.4** | **±** | **0.6** | **cd** | **0.5** | **±** | **0.5** | **ab** | **3.1** | **±** | **0.6** | **acd** |
| 14:1 | 0.0 | ± | 0.0 |  | 0.0 | ± | 0.0 |  | 0.0 | ± | 0.0 |  | 0.0 | ± | 0.0 |  | 0.0 | ± | 0.0 |  | 0.0 | ± | 0.0 |  | 0.4 | ± | 0.3 |  |
| **15:0** | **0.0** | **±** | **0.0** | **b** | **0.0** | **±** | **0.0** | **b** | **0.0** | **±** | **0.0** | **b** | **0.0** | **±** | **0.0** | **b** | **0.0** | **±** | **0.0** | **b** | **1.3** | **±** | **0.8** | **a** | **0.0** | **±** | **0.0** | **b** |
| a15:0 | 0.0 | ± | 0.0 |  | 0.0 | ± | 0.0 |  | 0.4 | ± | 0.4 |  | 0.3 | ± | 0.3 |  | 0.0 | ± | 0.0 |  | 0.0 | ± | 0.0 |  | 1.0 | ± | 0.4 |  |
| i15:0 | 0.0 | ± | 0.0 |  | 0.0 | ± | 0.0 |  | 0.9 | ± | 0.4 |  | 0.0 | ± | 0.0 |  | 3.4 | ± | 1.9 |  | 0.7 | ± | 0.7 |  | 2.0 | ± | 0.5 |  |
| **16:0** | **17.9** | **±** | **0.5** | **bc** | **22.6** | **±** | **1.4** | **bc** | **16.1** | **±** | **2.3** | **bc** | **9.6** | **±** | **7.9** | **a** | **8.9** | **±** | **1.4** | **ab** | **15.2** | **±** | **4.6** | **abc** | **19.8** | **±** | **1.4** | **bc** |
| 16:1ω5 | 0.0 | ± | 0.0 |  | 0.0 | ± | 0.0 |  | 0.0 | ± | 0.0 |  | 0.0 | ± | 0.0 |  | 0.0 | ± | 0.0 |  | 0.0 | ± | 0.0 |  | 0.0 | ± | 0.0 |  |
| **16:1ω7** | **0.6** | **±** | **0.3** | **b** | **0.5** | **±** | **0.2** | **b** | **0.6** | **±** | **0.3** | **b** | **0.8** | **±** | **0.8** | **b** | **1.8** | **±** | **0.4** | **bc** | **8.3** | **±** | **1.5** | **a** | **5.7** | **±** | **1.3** | **ac** |
| i16:0 | 0.0 | ± | 0.0 |  | 0.0 | ± | 0.0 |  | 0.0 | ± | 0.0 |  | 0.0 | ± | 0.0 |  | 0.0 | ± | 0.0 |  | 0.0 | ± | 0.0 |  | 0.3 | ± | 0.2 |  |
| 17:0 | 0.0 | ± | 0.0 |  | 0.0 | ± | 0.0 |  | 0.0 | ± | 0.0 |  | 0.7 | ± | 0.7 |  | 0.0 | ± | 0.0 |  | 0.0 | ± | 0.0 |  | 0.6 | ± | 0.4 |  |
| 17:1ω8 | 0.0 | ± | 0.0 |  | 0.0 | ± | 0.0 |  | 0.2 | ± | 0.2 |  | 1.3 | ± | 1.3 |  | 0.0 | ± | 0.0 |  | 0.0 | ± | 0.0 |  | 0.2 | ± | 0.2 |  |
| i17:0 | 0.0 | ± | 0.0 |  | 0.0 | ± | 0.0 |  | 0.0 | ± | 0.0 |  | 6.8 | ± | 6.8 |  | 0.0 | ± | 0.0 |  | 0.4 | ± | 0.4 |  | 0.6 | ± | 0.4 |  |
| cy17:0 | 0.0 | ± | 0.0 |  | 0.0 | ± | 0.0 |  | 0.2 | ± | 0.2 |  | 0.0 | ± | 0.0 |  | 1.3 | ± | 0.0 |  | 0.0 | ± | 0.0 |  | 0.2 | ± | 0.2 |  |
| **18:0** | **9.0** | **±** | **0.6** | **abc** | **12.7** | **±** | **1.5** | **bc** | **6.2** | **±** | **3.3** | **ac** | **7.3** | **±** | **7.3** | **ac** | **0.9** | **±** | **0.9** | **a** | **5.1** | **±** | **2.7** | **abc** | **15.8** | **±** | **0.9** | **b** |
| **18:1ω7** | **6.7** | **±** | **0.9** | **c** | **5.7** | **±** | **1.7** | **bc** | **0.0** | **±** | **0.0** | **a** | **1.1** | **±** | **0.8** | **a** | **0.0** | **±** | **0.0** | **a** | **1.0** | **±** | **0.5** | **ab** | **1.5** | **±** | **0.8** | **a** |
| **18:1ω9** | **34.6** | **±** | **0.8** | **a** | **29.0** | **±** | **1.8** | **a** | **32.4** | **±** | **3.1** | **a** | **26.5** | **±** | **7.6** | **ab** | **41.9** | **±** | **2.1** | **a** | **34.8** | **±** | **11.7** | **a** | **12.4** | **±** | **3.1** | **b** |
| **18:2ω6,9** | **19.3** | **±** | **0.2** | **abcd** | **14.6** | **±** | **1.2** | **acd** | **37.6** | **±** | **3.8** | **b** | **34.9** | **±** | **11.1** | **ab** | **32.3** | **±** | **2.2** | **ab** | **25.4** | **±** | **1.5** | **abc** | **12.4** | **±** | **1.7** | **cd** |
| 19:0 | 0.0 | ± | 0.0 |  | 0.0 | ± | 0.0 |  | 1.2 | ± | 0.8 |  | 0.4 | ± | 0.4 |  | 0.0 | ± | 0.0 |  | 0.0 | ± | 0.0 |  | 0.3 | ± | 0.3 |  |
| cy19:0 | 0.0 | ± | 0.0 |  | 0.0 | ± | 0.0 |  | 1.0 | ± | 0.6 |  | 1.7 | ± | 1.7 |  | 0.0 | ± | 0.0 |  | 0.0 | ± | 0.0 |  | 1.0 | ± | 0.5 |  |
| 20:1ω9 | 0.3 | ± | 0.3 |  | 0.1 | ± | 0.1 |  | 0.0 | ± | 0.0 |  | 0.0 | ± | 0.0 |  | 0.5 | ± | 0.5 |  | 1.7 | ± | 0.9 |  | 0.5 | ± | 0.3 |  |
| 20:2ω6,9 | 0.6 | ± | 0.3 |  | 0.2 | ± | 0.2 |  | 0.0 | ± | 0.0 |  | 0.0 | ± | 0.0 |  | 0.0 | ± | 0.0 |  | 0.7 | ± | 0.7 |  | 0.5 | ± | 0.3 |  |
| 20:3ω6 | 0.0 | ± | 0.0 |  | 0.0 | ± | 0.0 |  | 0.0 | ± | 0.0 |  | 0.0 | ± | 0.0 |  | 0.0 | ± | 0.0 |  | 0.0 | ± | 0.0 |  | 0.7 | ± | 0.4 |  |
| **20:4ω6** | **4.6** | **±** | **0.6** | **a** | **3.8** | **±** | **1.0** | **a** | **0.0** | **±** | **0.0** | **c** | **0.0** | **±** | **0.0** | **bc** | **0.0** | **±** | **0.0** | **bc** | **4.1** | **±** | **0.4** | **a** | **4.9** | **±** | **0.9** | **a** |
| **20:5ω3** | **6.1** | **±** | **0.6** | **def** | **8.1** | **±** | **1.2** | **ef** | **0.0** | **±** | **0.0** | **a** | **0.0** | **±** | **0.0** | **ab** | **0.0** | **±** | **0.0** | **ab** | **0.8** | **±** | **0.4** | **abc** | **11.6** | **±** | **2.5** | **f** |
| **22:1ω9** | **0.0** | **±** | **0.0** | **ab** | **0.0** | **±** | **0.0** | **a** | **0.3** | **±** | **0.3** | **ab** | **0.0** | **±** | **0.0** | **ab** | **0.0** | **±** | **0.0** | **ab** | **0.0** | **±** | **0.0** | **ab** | **1.3** | **±** | **0.7** | **b** |
| 22:2 | 0.0 | ± | 0.0 |  | 0.2 | ± | 0.2 |  | 0.0 | ± | 0.0 |  | 0.0 | ± | 0.0 |  | 0.0 | ± | 0.0 |  | 0.0 | ± | 0.0 |  | 1.0 | ± | 0.5 |  |
| **23:0** | **0.0** | **±** | **0.0** | **a** | **0.2** | **±** | **0.2** | **a** | **0.0** | **±** | **0.0** | **a** | **3.2** | **±** | **1.9** | **b** | **0.4** | **±** | **0.4** | **ab** | **0.0** | **±** | **0.0** | **a** | **0.0** | **±** | **0.0** | **a** |
| 24:1 | 0.0 | ± | 0.0 |  | 0.0 | ± | 0.0 |  | 0.2 | ± | 0.2 |  | 0.0 | ± | 0.0 |  | 1.5 | ± | 0.2 |  | 0.0 | ± | 0.0 |  | 0.0 | ± | 0.0 |  |

| **Table S3** |  |  |  |  |  |  |  |
| --- | --- | --- | --- | --- | --- | --- | --- |
| ANOVA table of logit-transformed proportions for each fatty acid from 13 field-collected Collembola species. Species and habitats are used as explanatory factors and sites and habitats as error terms in the model. *P*-values were obtained after Holm’s correction. Fatty acids significantly different between species are marked in bold. | | | | | | | |
| Fatty acid | Species | | |  | Habitat:Species | | |
|  | df | F | Adjusted *P* |  | df | F | Adjusted *P* |
| 8:0 | 12 | 2.111 | 0.539 |  | 8 | 1.077 | 0.975 |
| 10:0 | 12 | 1.622 | 1.000 |  | 8 | 1.083 | 0.975 |
| 2-OH 10:0 | 12 | 2.050 | 0.539 |  | 8 | 2.288 | 0.211 |
| 12:0 | 12 | 0.182 | 1.000 |  | 8 | 0.235 | 1.000 |
| **14:0** | **12** | **5.324** | **0.001** |  | 8 | 2.063 | 0.277 |
| 14:1 | 12 | 2.258 | 0.423 |  | 8 | 2.520 | 0.193 |
| **15:0** | **12** | **4.630** | **0.002** |  | 8 | 0.000 | 1.000 |
| a15:0 | 12 | 1.364 | 1.000 |  | 8 | 1.895 | 0.340 |
| i15:0 | 12 | 2.894 | 0.100 |  | 8 | 1.331 | 0.738 |
| **16:0** | **12** | **4.759** | **0.002** |  | 8 | 0.214 | 1.000 |
| 16:1ω5 | 12 | 1.336 | 1.000 |  | 8 | 0.091 | 1.000 |
| **16:1ω7** | **12** | **6.120** | **< 0.001** |  | 8 | 1.470 | 0.626 |
| i16:0 | 12 | 0.558 | 1.000 |  | 8 | 0.629 | 1.000 |
| 17:0 | 12 | 0.954 | 1.000 |  | 8 | 0.934 | 1.000 |
| 17:1ω8 | 12 | 1.135 | 1.000 |  | 8 | 0.623 | 1.000 |
| i17:0 | 12 | 0.894 | 1.000 |  | 8 | 1.003 | 1.000 |
| cy17:0 | 12 | 2.150 | 0.521 |  | 8 | 1.660 | 0.482 |
| **18:0** | **12** | **3.657** | **0.018** |  | 8 | 0.901 | 1.000 |
| **18:1ω7** | **12** | **4.975** | **0.001** |  | 8 | 0.855 | 1.000 |
| **18:1ω9** | **12** | **4.761** | **0.002** |  | 8 | 0.420 | 1.000 |
| **18:2ω6,9** | **12** | **6.162** | **< 0.001** |  | 8 | 0.705 | 1.000 |
| 19:0 | 12 | 2.571 | 0.214 |  | 8 | 2.514 | 0.193 |
| cy19:0 | 12 | 2.508 | 0.237 |  | 8 | 2.272 | 0.211 |
| 20:1ω9 | 12 | 2.062 | 0.539 |  | 8 | 0.278 | 1.000 |
| 20:2ω6,9 | 12 | 0.962 | 1.000 |  | 8 | 0.087 | 1.000 |
| 20:3ω6 | 12 | 0.734 | 1.000 |  | 8 | 0.272 | 1.000 |
| **20:4ω6** | **12** | **8.411** | **< 0.001** |  | 8 | 0.354 | 1.000 |
| **20:5ω3** | **12** | **16.549** | **< 0.001** |  | 8 | 0.354 | 1.000 |
| **22:1ω9** | **12** | **3.519** | **0.023** |  | 8 | 2.538 | 0.193 |
| 22:2 | 12 | 0.688 | 1.000 |  | 8 | 0.043 | 1.000 |
| **23:0** | **12** | **3.663** | **0.018** |  | 8 | 2.642 | 0.193 |
| 24:1 | 12 | 2.109 | 0.539 |  | 8 | 0.493 | 1.000 |

| **Table S4** | | | | | | | | | | | |
| --- | --- | --- | --- | --- | --- | --- | --- | --- | --- | --- | --- |
| Correlations of different fatty acids with the first four axes (PCs) from principle component analyses. *P*-values were adjusted using Holm’s method. Fatty acids correlated with the first PC are marked in bold. | | | | | | | | | | | |
|  | PC1 | |  | PC2 | |  | PC3 | |  | PC4 | |
|  | *cor | *P* |  | cor | *P* |  | cor | *P* |  | cor | *P* |
| *Field-derived species* |  |  |  |  |  |  |  |  |  |  |  |
| 8:0 | 0.483 | 1.000 |  | 0.121 | 1.000 |  | -0.225 | 1.000 |  | 0.649 | 0.476 |
| 10:0 | -0.566 | 1.000 |  | 0.036 | 1.000 |  | -0.575 | 0.832 |  | 0.248 | 1.000 |
| 2-OH 10:0 | -0.404 | 1.000 |  | 0.224 | 1.000 |  | -0.697 | 0.220 |  | -0.121 | 1.000 |
| 12:0 | -0.402 | 1.000 |  | 0.013 | 1.000 |  | -0.744 | 0.107 |  | 0.141 | 1.000 |
| 14:0 | -0.117 | 1.000 |  | **-0.850** | **0.008** |  | -0.361 | 1.000 |  | 0.074 | 1.000 |
| 14:1 | -0.404 | 1.000 |  | 0.224 | 1.000 |  | -0.697 | 0.220 |  | -0.121 | 1.000 |
| 15:0 | 0.161 | 1.000 |  | 0.151 | 1.000 |  | -0.125 | 1.000 |  | -0.770 | 0.065 |
| a15:0 | -0.259 | 1.000 |  | 0.103 | 1.000 |  | -0.656 | 0.360 |  | 0.277 | 1.000 |
| i15:0 | 0.175 | 1.000 |  | -0.218 | 1.000 |  | -0.704 | 0.203 |  | -0.130 | 1.000 |
| **16:0** | **-0.778** | **0.048** |  | -0.235 | 1.000 |  | 0.422 | 1.000 |  | -0.178 | 1.000 |
| 16:1ω5 | -0.185 | 1.000 |  | -0.103 | 1.000 |  | -0.013 | 1.000 |  | 0.202 | 1.000 |
| 16:1ω7 | -0.095 | 1.000 |  | 0.033 | 1.000 |  | -0.547 | 1.000 |  | -0.751 | 0.092 |
| i16:0 | -0.495 | 1.000 |  | -0.000 | 1.000 |  | -0.627 | 0.478 |  | 0.077 | 1.000 |
| 17:0 | 0.073 | 1.000 |  | 0.413 | 1.000 |  | -0.669 | 0.311 |  | 0.352 | 1.000 |
| 17:1ω8 | 0.237 | 1.000 |  | 0.238 | 1.000 |  | -0.263 | 1.000 |  | 0.538 | 1.000 |
| i17:0 | 0.298 | 1.000 |  | 0.339 | 1.000 |  | -0.486 | 1.000 |  | 0.518 | 1.000 |
| cy17:0 | 0.484 | 1.000 |  | -0.354 | 1.000 |  | -0.321 | 1.000 |  | -0.073 | 1.000 |
| **18:0** | **-0.953** | **< 0.001** |  | 0.158 | 1.000 |  | -0.022 | 1.000 |  | 0.135 | 1.000 |
| 18:1ω7 | -0.357 | 1.000 |  | 0.674 | 0.358 |  | 0.252 | 1.000 |  | -0.003 | 1.000 |
| 18:1ω9 | 0.235 | 1.000 |  | -0.573 | 1.000 |  | 0.731 | 0.130 |  | -0.081 | 1.000 |
| **18:2ω6,9** | **0.849** | **0.007** |  | 0.372 | 1.000 |  | 0.087 | 1.000 |  | -0.231 | 1.000 |
| 19:0 | 0.382 | 1.000 |  | 0.178 | 1.000 |  | -0.182 | 1.000 |  | 0.225 | 1.000 |
| cy19:0 | 0.208 | 1.000 |  | 0.314 | 1.000 |  | -0.294 | 1.000 |  | 0.339 | 1.000 |
| 20:1ω9 | 0.122 | 1.000 |  | 0.195 | 1.000 |  | -0.331 | 1.000 |  | **-0.805** | **0.029** |
| 20:2ω6,9 | -0.294 | 1.000 |  | 0.516 | 1.000 |  | -0.243 | 1.000 |  | -0.584 | 0.973 |
| 20:3ω6 | -0.452 | 1.000 |  | 0.104 | 1.000 |  | **-0.814** | **0.022** |  | 0.047 | 1.000 |
| **20:4ω6** | **-0.854** | **0.006** |  | 0.037 | 1.000 |  | -0.115 | 1.000 |  | -0.276 | 1.000 |
| **20:5ω3** | **-0.948** | **< 0.001** |  | 0.181 | 1.000 |  | -0.019 | 1.000 |  | 0.069 | 1.000 |
| 22:1ω9 | -0.320 | 1.000 |  | 0.227 | 1.000 |  | -0.631 | 0.478 |  | -0.154 | 1.000 |
| 22:2 | -0.508 | 1.000 |  | 0.189 | 1.000 |  | -0.774 | 0.059 |  | 0.042 | 1.000 |
| 23:0 | 0.515 | 1.000 |  | 0.259 | 1.000 |  | -0.237 | 1.000 |  | 0.627 | 0.613 |
| 24:1 | 0.493 | 1.000 |  | -0.429 | 1.000 |  | -0.171 | 1.000 |  | -0.029 | 1.000 |
|  |  |  |  |  |  |  |  |  |  |  |  |
| *Literature 37 species* |  |  |  |  |  |  |  |  |  |  |  |
| a15:0 | -0.066 | 1.000 |  | **-0.618** | **0.001** |  | -0.355 | 0.336 |  | -0.027 | 1.000 |
| i15:0 | 0.148 | 1.000 |  | -0.240 | 1.000 |  | -0.300 | 0.569 |  | -0.246 | 0.878 |
| 16:0 | -0.256 | 1.000 |  | **-0.870** | **< 0.001** |  | -0.040 | 1.000 |  | -0.181 | 1.000 |
| i16:0 | -0.215 | 1.000 |  | **-0.461** | **0.045** |  | -0.219 | 1.000 |  | 0.290 | 0.820 |
| 16:1ω7 | 0.207 | 1.000 |  | 0.029 | 1.000 |  | **0.531** | **0.010** |  | 0.351 | 0.400 |
| i17:0 | 0.150 | 1.000 |  | 0.028 | 1.000 |  | -0.214 | 1.000 |  | -0.189 | 1.000 |
| **18:0** | **-0.643** | **< 0.001** |  | **-0.481** | **0.031** |  | -0.046 | 1.000 |  | -0.112 | 1.000 |
| **18:1ω7** | **-0.825** | **< 0.001** |  | -0.063 | 1.000 |  | -0.252 | 0.932 |  | 0.394 | 0.224 |
| **18:1ω9** | **0.477** | **0.031** |  | 0.183 | 1.000 |  | **0.586** | **0.002** |  | -0.283 | 0.820 |
| **18:2ω6,9** | **0.542** | **0.006** |  | **0.572** | **0.003** |  | **-0.513** | **0.014** |  | 0.256 | 0.878 |
| cy19:0 | 0.110 | 1.000 |  | 0.326 | 0.441 |  | -0.311 | 0.551 |  | -0.180 | 1.000 |
| 20:1ω9 | 0.052 | 1.000 |  | -0.163 | 1.000 |  | **0.523** | **0.012** |  | **0.526** | **0.013** |
| 20:2ω6,9 | -0.136 | 1.000 |  | -0.002 | 1.000 |  | -0.356 | 0.336 |  | 0.327 | 0.532 |
| 20:3ω6 | 0.343 | 0.379 |  | 0.071 | 1.000 |  | **0.712** | **< 0.001** |  | 0.277 | 0.820 |
| **20:4ω6** | **-0.649** | **< 0.001** |  | 0.344 | 0.370 |  | 0.182 | 1.000 |  | 0.427 | 0.125 |
| **20:5ω3** | **-0.696** | **< 0.001** |  | **0.562** | **0.004** |  | 0.168 | 1.000 |  | -0.393 | 0.224 |
| * Pearson correlation coefficient | | | | | | | | | | | |

| **Table S5** |  |  |  |  |  |  |  |  |  |  |  |
| --- | --- | --- | --- | --- | --- | --- | --- | --- | --- | --- | --- |
| ANOVA table of logit-transformed proportions for each fatty acid in the expanded 37-species dataset. Species and habitats are used as explanatory factors in the model. *P*-values were obtained after Holm’s correction. Fatty acids significantly different between species are marked in bold. | | | | | | | | | | | |
| Fatty acid | Habitat | | |  | Species | | |  | Habitat:Species | | |
|  | df | F | Adjusted *P* |  | df | F | Adjusted *P* |  | df | F | Adjusted *P* |
| a15:0 | 2 | 3.090 | 0.327 |  | 36 | 0.877 | 1.000 |  | 9 | 0.415 | 1.000 |
| **i15:0** | 2 | 2.899 | 0.327 |  | **36** | **2.146** | **0.011** |  | 9 | 0.811 | 1.000 |
| **16:0** | **2** | **23.017** | **< 0.001** |  | **36** | **3.557** | **< 0.001** |  | 9 | 1.065 | 1.000 |
| **16:1ω7** | 2 | 1.545 | 0.825 |  | **36** | **2.370** | **0.004** |  | 9 | 0.347 | 1.000 |
| i16:0 | 2 | 3.160 | 0.327 |  | 36 | 1.422 | 0.440 |  | 9 | 0.192 | 1.000 |
| i17:0 | 2 | 0.535 | 0.825 |  | 36 | 0.716 | 1.000 |  | 9 | 0.845 | 1.000 |
| **18:0** | **2** | **15.104** | **< 0.001** |  | **36** | **2.619** | **0.001** |  | **9** | **3.424** | **0.016** |
| 18:1ω7 | **2** | **35.324** | **< 0.001** |  | 36 | 1.522 | 0.319 |  | 9 | 0.693 | 1.000 |
| **18:1ω9** | **2** | **11.782** | **< 0.001** |  | **36** | **2.278** | **0.006** |  | 9 | 1.174 | 1.000 |
| **18:2ω6,9** | **2** | **31.610** | **< 0.001** |  | **36** | **3.176** | **< 0.001** |  | 9 | 0.918 | 1.000 |
| cy19:0 | **2** | **12.049** | **< 0.001** |  | 36 | 1.379 | 0.440 |  | 9 | 1.191 | 1.000 |
| **20:1ω9** | **2** | **40.593** | **< 0.001** |  | **36** | **3.760** | **< 0.001** |  | 9 | 2.943 | 0.054 |
| 20:2ω6,9 | 2 | 1.603 | 0.825 |  | 36 | 0.841 | 1.000 |  | 9 | 0.103 | 1.000 |
| **20:3ω6** | **2** | **87.090** | **< 0.001** |  | **36** | **3.637** | **< 0.001** |  | **9** | **3.016** | **0.047** |
| **20:4ω6** | **2** | **5.363** | **0.049** |  | **36** | **4.431** | **< 0.001** |  | 9 | 1.072 | 1.000 |
| **20:5ω3** | 2 | 1.244 | 0.825 |  | **36** | **3.418** | **< 0.001** |  | 9 | 1.961 | 0.671 |
