# Supplementary material for: Neutral lipid fatty acid composition as trait and constraint in Collembola evolution
Source: Ecol Evol. 2017 Oct 16;7(22):9624–38. doi: 10.1002/ece3.3472 (PMC5696395; doi:10.1002/ece3.3472)
Supplement: Supplementary file 3 [file ECE3-7-9624-s003.doc]

**Supplementary Materials**

A phylogeny of the 37 Collembola species was constructed using six genetic markers: 18S rRNA, 28S rRNA D1, D2 and D3 regions, cytochrome oxidase subunit I (COI) and Histone H3 genes. *Callibaetis* (Insecta: Ephemeroptera), *Machilis* (Insecta: Archaeognatha) and Zygentoma (Insecta: Zygentoma) were used as outgroups. Sequences were downloaded from GenBank ([www.ncbi.nih.gov](http://www.ncbi.nih.gov/); Accession Number see Table S1). Species without sequence data available in GenBank were replaced by the taxonomically closest species, usually a congener (Table 1). The six genetic markers were aligned separately in R 3.2.2 (R Core Team 2015) using functions *AlignSeqs* and *AdjustAlignment* for 18S and 28S rRNA and Histone H3 genes (package “DECIPHER”; Wright 2015) and function *msaClustalW* for COI by setting gap opening as 15 and gap extension as 6.6 (package “msa”; Bodenhofer *et al.* 2015). The aligned sequences were trimmed to the same length in BioEdit 7.2.5 (Hall 1999). Models of sequence evolution for each marker were obtained using jModelTest 2.1.4 and based on the Alkaike information criterion (Darriba *et al.* 2012). Terminal gaps in each marker set were replaced by “?” and the six markers were concatenated in a supermatrix (3,053 bp) using SequenceMatrix 1.8 (Vaidya, Lohman & Meier 2011). The phylogeny was inferred using Bayesian Inference (BI) in MrBayes 3.2.4 (Ronquist *et al.* 2012), setting models of sequence evolution for each marker separately as suggested by jModelTest. Bayesian Inference was conducted using two independent runs of four chains for 1,000,000 generations and the consensus tree generated using a burn-in of 0.25. A second phylogenetic tree was constructed using Maximum Likelihood (ML) in RAxML 7.0.3 (Stamatakis 2006) based on the GTR+I+G model and 1000 bootstrap replicates. The topologies of the phylogenetic trees of BI and ML were similar, except for the sister taxon of Poduromorpha. In the BI tree, Tomoceridae was sister of Poduromorpha (Figure S1), while in the ML tree it was Symphypleona (Figure S2). The BI tree was selected and transformed to an ultrametric tree using a penalized likelihood approach assuming different models of substitution rate variation among branches, including correlated, relaxed, discrete or strict clock models, using the function *chronos* implemented in the R package “ape” (Paradis, Claude & Strimmer 2004). The ultrametric tree for downstream phylogenetic signal analyses was selected based on the smallest PHIIC value, a criterion analogous to Alkaike information criterion reflecting the best model fit to the data (Paradis 2013). Concomitantly, a strict clock model was used in the phylogenetic analyses.

**Literature Cited**

Bodenhofer, U., Bonatesta, E., Horejs-Kainrath, C. & Hochreiter, S. (2015) msa: An R package for multiple sequence alignment. *Bioinformatics*, **31**, 3997–3999.

Darriba, D., Taboada, G.L., Doallo, R. & Posada, D. (2012) jModelTest 2: more models, new heuristics and parallel computing. *Nature Methods*, **9**, 772.

Hall, T.A. (1999) BioEdit: a user-friendly biological sequence alignment editor and analysis program for Windows 95/98/NT. *Nucleic Acids Symposium Series*, **41**, 95–98.

Paradis, E., Claude, J. & Strimmer, K. (2004) APE: Analyses of phylogenetics and evolution in R language. *Bioinformatics*, **20**, 289–290.

Paradis, E. (2013) Molecular dating of phylogenies by likelihood methods: A comparison of models and a new information criterion. *Molecular Phylogenetics and Evolution*, **67**, 436-444.

R Core Team. (2015) R: A language and environment for statistical computing.

Ronquist, F., Teslenko, M., Van Der Mark, P., Ayres, D.L., Darling, A., Höhna, S., Larget, B., Liu, L., Suchard, M.A. & Huelsenbeck, J.P. (2012) Mrbayes 3.2: Efficient bayesian phylogenetic inference and model choice across a large model space. *Systematic Biology*, **61**, 539–542.

Stamatakis, A. (2006) RAxML-VI-HPC: Maximum likelihood-based phylogenetic analyses with thousands of taxa and mixed models. *Bioinformatics*, **22**, 2688–2690.

Vaidya, G., Lohman, D.J. & Meier, R. (2011) SequenceMatrix: Concatenation software for the fast assembly of multi-gene datasets with character set and codon information. *Cladistics*, **27**, 171–180.

Wright, E.S. (2015) DECIPHER: harnessing local sequence context to improve protein multiple sequence alignment. *BMC Bioinformatics*, **16**, 322.

**Legends to supplementary figures**

**Figure S1**

Bayesian Inference phylogeny of Collembola based on the concatenated DNA sequences of ribosomal 18S and 28S rRNA and cytochrome oxidase I and Histone H3 genes. *Callibaetis*, *Machilis* and Zygentoma served as outgroups. Numbers at nodes represent Bayesian posterior probabilities.

**Figure S2**

Maximum likelihood phylogeny of Collembola based on the concatenated DNA sequences of ribosomal 18S and 28S rRNA and cytochrome oxidase I and Histone H3 genes. *Callibaetis*, *Machilis* and Zygentoma served as outgroups. Numbers at nodes represent bootstrap values from Maximum Likelihood analyses.
